# In vitro influence of PEG functionalized ZnO–CuO nanocomposites on bacterial growth

**DOI:** 10.1038/s41598-024-52014-6

**Published:** 2024-01-14

**Authors:** Madara Jayanetti, Charitha Thambiliyagodage, Heshan Liyanaarachchi, Geethma Ekanayake, Amavin Mendis, Leshan Usgodaarachchi

**Affiliations:** Faculty of Humanities and Sciences, Sri Lanka Institute of Information Technology, New Kandy Road, Malabe, Sri Lanka

**Keywords:** Biotechnology, Chemistry, Nanoscience and technology

## Abstract

Polyethyleneglycol-coated biocompatible CuO–ZnO nanocomposites were fabricated hydrothermally varying Zn:Cu ratios as 1:1, 2:1, and 1:2, and their antibacterial activity was determined through the well diffusion method against the Gram-negative *Escherichia coli****,**** Pseudomonas aeruginosa*, *Klebsiella pneumoniae,* and the Gram-positive *Staphylococcus aureus.* The minimum inhibitory concentration and the minimum bactericidal concentration values of the synthesized samples were determined. Subsequently, the time synergy kill assay was performed to elucidate the nature of the overall inhibitory effect against the aforementioned bacterial species. The mean zone of inhibition values for all four samples are presented. The inhibitory effect increased with increasing concentration of the nanocomposite (20, 40 and 60 mg/ml) on all the bacterial species except for *S. aureus*. According to the MBC/MIC ratio, ZnO was found to be bacteriostatic for *E. coli* and *P. aeruginosa,* and bactericidal for *S. aureus* and *K. pneumoniae*. Zn:Cu 2:1 was bactericidal on all bacterial species. A bacteriostatic effect was observed on *E. coli* and *P. aeruginosa* in the presence of Zn:Cu 1:1 whereas, it showed a bactericidal effect on *S. aureus* and *K. pneumoniae.* Zn:Cu 1:2 exhibited a bacteriostatic effect on *E. coli* while a bactericidal effect was observed for *E. coli, P. aeruginosa,* and *K. pneumoniae.* The metal oxide nanocomposites were found to be more sensitive towards the Gram-positive strain than the Gram-negative strains*.* Further, all the nanocomposites possess anti-oxidant activity as shown by the DPPH assay.

## Introduction

The development of new and effective substances against pathological bacteria is receiving a great deal of attention since there is a number of concerns about bacterial resistance to various current antibiotics or disinfectants^1^. Although antimicrobial agent resistance has been known for more than 50 years, it is still a significant factor in rising morbidity, death, and medical expense^2^. The overuse of antibiotics is thought to be the primary contributing factor, but inadequate infection control methods, extended hospital stays, admission to intensive care units, the use of invasive operations, and environmental discharge of antimicrobial substances without regulation are all contributors^2^. The key challenges of antibiotic therapy include overuse and misuse, multi-drug resistance, side effects, potential allergies, improper administration and the lack of new developments to treat infections (Ref). Even though improvements in the field of antibiotics have increased the average human lifetime, there are challenges which require novel and advanced solutions. Finding novel chemical materials with the distinct physicochemical properties needed for the synthesis of antibiotics is a major scientific challenge. Drug resistance can be described as a state of insensitivity or decreased sensitivity to drugs that ordinarily cause growth inhibition or cell death. Bacteria, fungi, viruses, and particularly parasites all exhibit high levels of antimicrobial resistance and such resistance could be innate or acquired^3^. *Pseudomonas aeruginosa*, *Acinetobacter baumannii*, and Gram-negative bacilli that produce carbapenemase are among the organisms that cause infections that may be extensively drug-resistant (XDR) or pan drug-resistant (PDR). Moreover, all FDA-approved antibacterial drugs, except for aminoglycosides, tigecycline, and polymyxins B or E, are resistant to gram-negative bacilli (GNB)^4^. However, the focus of this work is on bacterial resistance and possible solutions for it. Gram-negative bacteria are resistant to glycopeptides, while Gram-positive bacteria are resistant to aztreonams. Anaerobic microorganisms, including *Enterococcus* species, are resistant to aminoglycosides while *Pseudomonas* species are resistant to tetracycline and penicillin except ureidopenicillins. *Staphylococcus* species are found to be resistant to Penicillin. The global threat posed by pneumococcal resistance keeps growing, as it began with penicillin resistance and now displays resistance to macrolides and tetracyclines^5^.

Therefore, it is of great importance to investigate alternative antibiotics which would be more active against broad-spectrum bacteria and cause fewer side effects along with no harm to the environment. Despite the new antibiotics being researched nanomaterials have attracted attention due to their high efficiency and effectiveness towards a broad range of bacterial species. Due to their size and capacity to damage cells through a variety of methods, nanoparticles have demonstrated antibacterial effectiveness towards a variety of diseases. Nanomaterials offer an intriguing way to restrict microbial development before human infection, in contrast to antibiotics, which are used to treat illnesses and infections in patients^6^. Nanostructure materials have been subjected to extensive research over the past ten years due to their unique physicochemical and biological properties^7^. This technology can be used for a range of novel applications, including cutting-edge fabric chemicals, waste water management, advanced pharmaceutical procedures, and food and agricultural production^8–10^. Due to their numerous bactericidal capabilities, straightforward production processes, good photo-responsive performance, etc., noble metal nanoparticles (NMNPs), particularly gold (Au), silver (Ag), and platinum (Pt), have drawn significant attention in the antimicrobial field^11,12^. Graphene oxide (GO) nanoparticles and other carbon-based nanomaterials, including fullerenes, carbon nanotubes (CNTs), particularly single-walled carbon nanotubes (SWCNTs), and carbon nanotubes (CNTs), have been found to exhibit strong antibacterial characteristics in recent studies^13^. Metal-based nanomaterials, such as Al_2_O_3_, CrO_3_, Fe_3_O_4_, SiO_2_, TiO_2_ and ZnO_2_ as well as quantum dots and different metallic nanoparticles like Ag, Au, and Pt. Such metal oxides have been discovered to be the underlying cause of conditions like oxidative stress, endothelial cell inflammation, apoptosis, and ecotoxicity^14^ It has been discovered that nanomaterials come in a wide variety of shapes and structures such as spheres, plates, tubes, needles, sheets, etc. which can influence the overall antibacterial capacity^15^. On the antibacterial characteristics of metal oxide nanoparticles, there is scarce information available compared to existing publications on chemical properties^16^.

ZnO in the nanoscale range has a wide range of forms and exhibits strong antibacterial activity against a wide range of bacterial species that have been researched extensively^17,18^. Due to their increased specific surface area and reduced particle size, which increases particle surface reactivity, ZnO-nanomaterials have appealing antibacterial capabilities^7,19,20^. ZnO doped with Boron and Zn doped CuO have been shown to act against *Staphylococcus aureus, Pseudomonas aeruginosa, Klebsiella pneumonia,* and *Escherichia coli*^21,22^. ZnO and ZnO coupled with Cu and CuO have not only been reported to be antibacterial but also have been photocatalytically active in degrading methylene blue which is important in wastewater treatment^23,24^.

Once within the bacterial cell, they interact with the surface and/or the core of the bacteria and exhibit specific bactericidal mechanisms^7,25^. CuO is another nano-metal oxide which has been researched for its ability to inhibit overall bacterial growth^25–28^ (add reference Cymbopogon citratus). Nanoflowers, nanorods, nanoleaves, and nanoflakes are examples of hierarchical cupric oxide (CuO) nanostructures which show significant antibacterial activity^29^. It is assumed that materials containing Cu nanoparticles are capable of killing both Gram-positive and Gram-negative bacteria through the "attract-kill-release" pathway^30^. Reactive oxygen species (ROS) generation and the release of Cu ions from Cu nanoparticles are both thought to be responsible for the contact killing of bacteria^30,31^. Additionally, they inactivate the microbes by promoting oxidative stress reactions, destroying membrane integrity and binding to the proteins^29,31–35^. PEGs identified as Macrogols, is a polyether of repeated ethylene glycol units [-(CH_2_CH_2_O)n]^36^ and it is renowned for their highly flexible structure, biocompatibility, amphiphilicity, lack of any steric obstructions, and high capacity for hydration^37^. PEGylation is known as the process of attaching one or more PEG molecules to substances used in treating or preventing disease, to modify the therapeutic efficacy^37,38^.

Numerous scientists have investigated the antioxidant capacity of several nanomaterials such as CeO_3_, Fe_3_O_4_, TiO_2_ and Se^6^. According to Saikia et al., nanoparticles of NiO and Fe_3_O_4_ exhibit strong antioxidant properties. Interestingly, CuO nanoparticles coated with polyethylene-glycol (PEG) and polyvinyl-pyrrolidone (PVP) have shown increased biological activities including antioxidant properties compared to the naked CuO nanoparticles^33^ whereas, CuO nanoparticles synthesized by thermal decomposition have shown effective antioxidant activity^35^. ZnO nanoparticles have also shown promising antioxidant activities. ZnO nanoparticles which were synthesized utilizing *Cassia sieberiana*'s methanolic root bark extract and the ZnO nanoparticles synthesized by *Pichia kudriavzevii* Yeast Strain have demonstrated potent antioxidant properties against the DPPH free radical scavenging assay^39^. However, the biocompatibility of those nanomaterials remains the challenge demarcating applicability of them in the biological systems. Hence, it is significant to determine a method of improving the biocompatibility of the nanomaterials to improve the overall effectiveness and efficiency.

In this study, we report the antibacterial activity and antioxidant activity by DPPH radical scavenging activity of PEG-coated ZnO–CuO nanocomposite on *Escherichia coli*, *Pseudomonas aeruginosa, Klebsiella pneumoniae* and *Staphylococcus aureus.* ZnO–CuO nanocomposites were functionalized with PEG to enhance the biocompatibility of the nanocomposite which increases the cell contact and cell uptake. The possible antibacterial mechanisms of PEG-coated ZnO and CuO nanomaterials are discussed in detail. To our knowledge, the antibacterial activity and the possible mechanisms of ZnO and CuO nanomaterials synthesized with PEG functionalization in the proposed proportions and with the co-precipitation method have not been reported.

## Materials and methodology

### Chemicals and materials

CuCl_2_ and ZnSO_4_ were procured from Sigma Aldrich (UK), NaOH pellets were purchased from Sisco Research Laboratories (Pvt) Ltd, India, PEG was purchased from HiMedia Leading Biosciences Company, Muller Hinton Agar was purchased from HiMedia Laboratories (Germany), Luria Bertani Broth (LB broth) was purchased from HiMedia Laboratories (Germany) and Deionized water (DI), with resistivity greater than 18.0 MΩ.cm (Millipore Milli-Q system), was used in the experiments. All of the chemicals utilized in the experiments were of analytical grade and were used without further purification.

### Synthesis of nanocomposites coated with PEG

Samples were synthesized in different ratios of ZnO and CuO as follows: ZnO: CuO, 1:1, 1:2, 2:1, and is expressed as pure ZnO, Zn: Cu(1:1), Zn: Cu(1:2) and Zn: Cu(2:1) in the text. Each metal salt was weighed to prepare the above-mentioned ratios and they were dissolved in a minimum amount of deionized water until a completely dissolved solution was obtained. For example, 16.147 g of ZnSO_4_ was dissolved and mixed with 15.961 g of CuSO_4_ to prepare Zn:Cu (1:1) composite. PEG powder (2 g) was dissolved in 100 ml of deionized water to prepare a 2% (w/w) solution. Then metal ion solutions were added dropwise to the PEG solution while stirring. Once the solution was homogenized in a sonicator for 30 min, 1% NaOH solution was added to the mixture dropwise and stirred for two hours until a dark blue precipitate appeared except for ZnSO_4_ where a white precipitate was obtained. Then stirring was continued overnight where a black colour precipitate was obtained except for pure ZnO. Then the solutions were hydrothermally treated in a hydrothermal via at 180 °C for 15 h. Then the obtained samples were filtered and washed with deionized water until the samples were free of Cl^−^ and SO_4_^2−^ ions, and a neutral pH was achieved. The washed samples were then oven-dried at 100 °C until completely dried and stored for further analysis.

### Antibacterial activity

#### Preparation of media

The required quantities of media were prepared with Muller Hinton agar and Luria Bertani broth using deionized water and sterilized in the autoclave.

#### Microbial strain and inoculum preparation

The test organisms, gram-negative *Escherichia coli*, *Pseudomonas aeruginosa*, *Klebsiella pneumoniae,* and gram-positive *Staphylococcus aureus*, were procured from Medical Research Institute, Sri Lanka. For preparing the inoculum, *Escherichia coli*, *Staphylococcus aureus*, *Pseudomonas aeruginosa*, and *Klebsiella pneumoniae* were cultured in Luria Bertani broth medium at 37 °C overnight. The microbial cultures were sub-cultured and overgrown 24 h prior to the assay and later diluted and adjusted the concentrations to obtain a microbial suspension of 5 × 10^5^ colony-forming units (CFUs)/ml using the spectrophotometer for further analysis^40,41^.

#### Agar well diffusion method

Nanocomposites were weighed (20, 40 and 60 mg), and sonicated to disperse in Dimethyl sulfoxide (DMSO) for 1 h. The Mueller Hinton Agar plate surface was inoculated by spreading the adjusted microbial inoculum of 5 × 10^5^ colony-forming units (CFUs)/ml over the entire agar surface via streaking. Holes wich were punched aseptically with a sterile cork borer and volume (70 µL) of the antimicrobial agent solution of desired concentrations; 20, 40 or 60 mg in 1 ml of DMSO was introduced into the wells^42^. A standard antibiotic (amoxicillin) and Dimethyl sulfoxide (DMSO) were also introduced into one well each as a positive and negative control, respectively. Then the agar plates were incubated at 37 °C for about 18 h and the zones of inhibition were measured in mm. Three replicates were prepared for each sample and each bacterial species^43^. The zone diameters were measured with the use of a metric ruler from the back of the Petri plate, while it was resting on a black, nonreflecting, flat surface, illuminated by a light source. Pairs of measurements were taken for each petri plate in mm and the average value was determined^44^. The antibacterial testing for PEG polymer was conducted via the agar well diffusion method as explained above. PEG polymer solutions of 20, 40 or 60 mg/ml concentrations were prepared by measuring the respective weights and dissolving them in DMSO solvent. Amoxicillin was used as a positive control while DMSO was used as a negative control in the agar well diffusion assay.

#### Minimum inhibitory (MIC) and minimum bactericidal concentration (MBC) evaluation

The antibacterial agents prepared were diluted into various concentrations, 0.3125, 0.625, 1.25, 2.5, 5, 10, 20, and 40 mg/ml, and a control concentration of 0 mg/ml in sterile Eppendorf tubes. Using a micropipette, 1 ml of each microbial culture was to be tested, and (0.5 McFarland standard) was inoculated into test tubes containing 2 ml of the various concentrations of the antibacterial agent in Luria Bertani broth for determination of MIC^45^. MBCs were determined by performing serial dilutions of the samples in DMSO and plated on to nutrient agar plates. In detail, 2 μL of the treated samples containing the nanomaterial and the test organism from each test tube, was inoculated into Muller Hinton agar plates for the determination of MBC.

No nanomaterial was introduced to the control Muller Hinton agar plate. Both the test tubes and the plates were then incubated at 37 °C for 18 to 24 h and thereafter observed for the growth of bacteria and viable count, respectively^46^. The minimum incubation concentration (MIC) of nanocomposite suspension was determined as the concentration at which there was no visible turbidity. In contrast, MBC was determined as the lowest concentration of nanocomposite suspension that prevented the growth of bacteria yielding three log reductions (99.9%) on spread plates^41^.

#### Time-kill synergy assay

To determine the antibacterial activity of the synthesized nanomaterials against the test bacterial pathogens, which were cultured at a concentration of 5 × 10^5^ colony-forming units (CFUs)/ml, 9 ml of LB broth and 1 ml of the nanosuspensions prepared were mixed. The time-kill synergy assay was carried out while the samples were kept in a rotary shaker at 200 rpm. The optical density was measured at intervals of 1 h for a total of 12 h using 600 nm wavelength. Turbidity was shown on a graph against time. To investigate for any indications of antibacterial actions of the synthesized nanomaterials, the growth curve thus obtained was examined. The positive control utilized was amoxicillin. The negative control was DMSO; the solvent used to dissolve the sample^47^.

The time-kill synergy assay was performed by the broth macro dilution method. Each pathogenic bacterial strain was tested against each nanocomposite. The time-kill assay was conducted with a final inoculum of approximately 5 × 10^5^ CFU/ml in a final volume of 10 ml. The concentration of the bacterial culture was verified with the spectrophotometer at 600 nm. LB broth and nano suspension from each sample were mixed in 9:1 ratio to obtain the 10 ml final volume in test tubes shaken continuously on an orbital shaker at 200 rpm and incubated at 37 °C. The optical density of each test tube was measured at intervals of 1 h for a total of 12 h using 600 nm wavelength. Turbidity was tabulated on a graph against time. To investigate the antibacterial actions of the synthesized nanomaterials, the growth curve obtained was examined. The positive control utilized was amoxicillin. The negative control was DMSO; the solvent used to dissolve the sample.

#### Determination of antioxidants by DPPH radical scavenging activity

The nanocomposites' free RSA was determined using the 1,1-diphenyl-2-picryl hydrazyl (DPPH) method. The stock solution was prepared by dissolving 24 mg of DPPH in 100 ml of methanol. The solution was filtered and used for the subsequent analysis. Methanol was used to prepare varied concentrations of synthesized nanomaterial ZnO, Zn:Cu 2:1, Zn:Cu 1:2 and Zn:Cu 1:1 (20 mg/ml). DPPH solution (500 ul) was mixed with 3 ml of the nanomaterial solution and incubated in the dark for 30 min which was exposed to sunlight after that. Absorbance was recorded at 517 nm for all three concentrations. 3 ml methanol mixed with 500 ul of DPPH solution was used as the positive control. The percentage (%) of inhibition was calculated to determine the antioxidant activity using the formula below:

$${\text{Scavenging}}\;{\text{effect}}\;\left( \% \right) = \left[ {\left( {{\text{Ac }} - {\text{ As}}} \right)/{\text{Ac}}} \right] \times {1}00$$

*Ac* absorbance of the control; *As* absorbance of the sample.

## Characterization

The XRD patterns were obtained using the D8 Advance Bruker machine, which employs Cu K α (λ = 0.154 nm) radiation, shifting the 2θ from 5° to 80° at a scan speed of 2°/min. The morphology of the produced nanocomposites was evaluated using transmission electron microscopy (TEM). The microscope was run at 200 kV (JEOL JEM 2100), and the energy-dispersive spectra (EDS) were also acquired by the same device with TEAM EDX software. Before the TEM investigation, a quantity of 1 L was put on a carbon copper grid with holes and let dry at room temperature. The EDAX element EDS system was used to capture the EDX spectra, while a ZEISS EVO 18 RESEARCH instrument was used to collect the SEM pictures. The survey spectra and higher-resolution spectra of the synthesized catalysts were collected using the Thermo Scientific ESCALAB Xi + X-ray photoelectron spectrometer. The Shimadzu 1800 UV–visible spectrophotometer, which uses a precise Czerny-Turner optical system, was used to gather the diffuse reflectance spectra of the prepared samples. With a bandwidth of 1.0 nm and a wavelength accuracy of ± 0.1 nm, measurements were made in the 400–750 nm range. The Raman analysis was performed using a Bruker Senterra Raman microscope spectrophotometer.

## Results

### FT-IR analysis

FT-IR spectra were collected to confirm the coating of PEG to the nanomaterials (supplementary Fig. 1). The C-O bond stretching frequency of aliphatic ether of PEG appeared at 1157 cm^−1^ and the C-H bending of the same appeared at 1458 cm^−1^ indicating the coating of PEG to the nanomaterials. Additionally, the peak at 2360 cm^−1^ is ascribed to the O = C = O of CO_2_ and the peaks in the range of 3600–3800 cm^−1^ are attributed to the stretching frequency of O–H.

### XRD analysis

**Figure 1:**
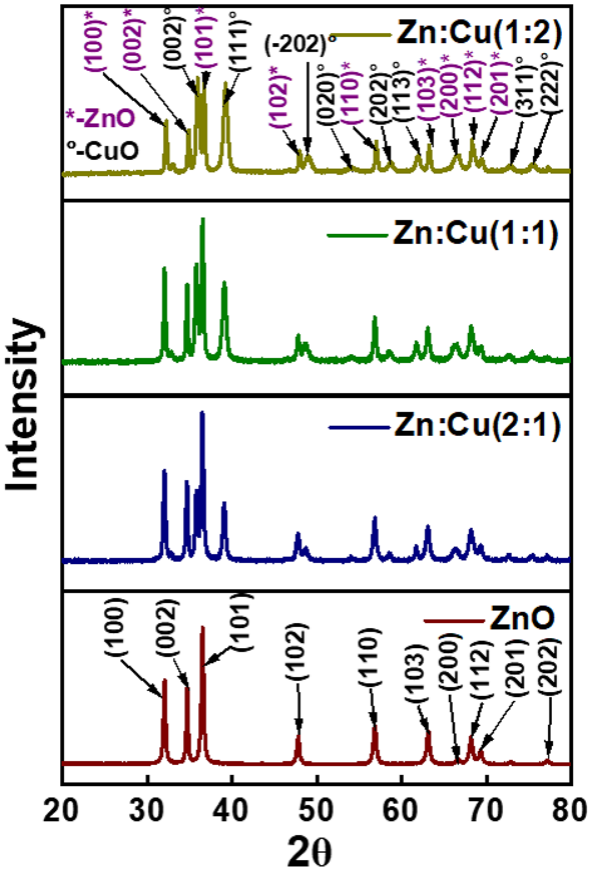
XRD patterns of the synthesized nanomaterials.

The XRD patterns were collected to determine the crystallographic orientation of the synthesized nanomaterials (Fig. 1). The XRD pattern of the ZnO nanomaterial consisted of the hexagonal wurtzite crystal structure. The pattern comprised of peaks at 2θ of 32.07, 34.74, 36.55, 47.83, 56.85, 63.13, 66.58, 68.19, 69.35, 73.26 and 77.70° which correspond to (100), (002), (101), (102), (110), (103), (200), (112), (201), (004) and (202) planes and the lattice constants were calculated to be a = b = 0.324 nm and c = 0.521 nm (JPCDS-36–141). The d spacing calculated by λ = 2dsinθ of the (101) plane represented by the peak at 36.55° is 0.2456 nm, the crystallite size calculated by the Debye–Scherrer formula (L = Kλ/β cosθ) is 48.50 nm and the lattice strain is 0.00238. XRD patterns of the composite materials consisted of peaks at 32.84, 35.84, 39.12, 48.64, 53.30, 61.75, 66.38 and 68.40, 72.40 and 75.16° additional to the peaks correspond to the ZnO phase and they are attributed to (110), (002), (111), (-202), (020), (-113), (-311), (220), (222) and (311) planes of monoclinic CuO (JCPDS-48–1548). The peak at 35.84° with the highest intensity was selected for further calculations. The interlayer distance of the (002) plane, the crystallite size and the lattice strain are calculated to be 0.2504 nm, 40.52 nm and 0.00290, respectively. The synthesized nanomaterials are composites of ZnO and CuO at different ratios. The respective parameters calculated from XRD data are tabulated in Table 1. The crystallite size of CuO decreased and the lattice strain increased with increasing weight ratio of CuO in the CuO–ZnO composite. However, the crystallite size of ZnO increased and the lattice strain decreased with increasing weight of CuO. Further, it is evident that the crystallographic orientation of the composite hasn’t been changed with increasing weight ratio of CuO in the composite and no other crystal natures of oxides of Cu were observed either. The composites are free of crystalline impurities as shown by the absence of peaks corresponding to such impurities in the XRD patterns.

**Table 1:** Crystallographic parameters of the nanomaterials.

Sample	Crystal plane	2θ (°)	L (nm)	d (nm)	L/d
ZnO	Zn (101)	36.548	48.57	0.2456	198
Zn:Cu (2:1)	Zn (101)	36.512	48.50	0.2459	198
	Cu (002)	35.833	49.23	0.2504	197
Zn:Cu (1:1)	Zn (101)	36.559	52.29	0.2456	213
	Cu (002)	35.836	40.53	0.2504	162
Zn:Cu(1:2)	Zn (101)	36.727	52.63	0.2445	215
	Cu (002)	35.978	36.17	0.2494	145

### SEM analysis

**Figure 2:**
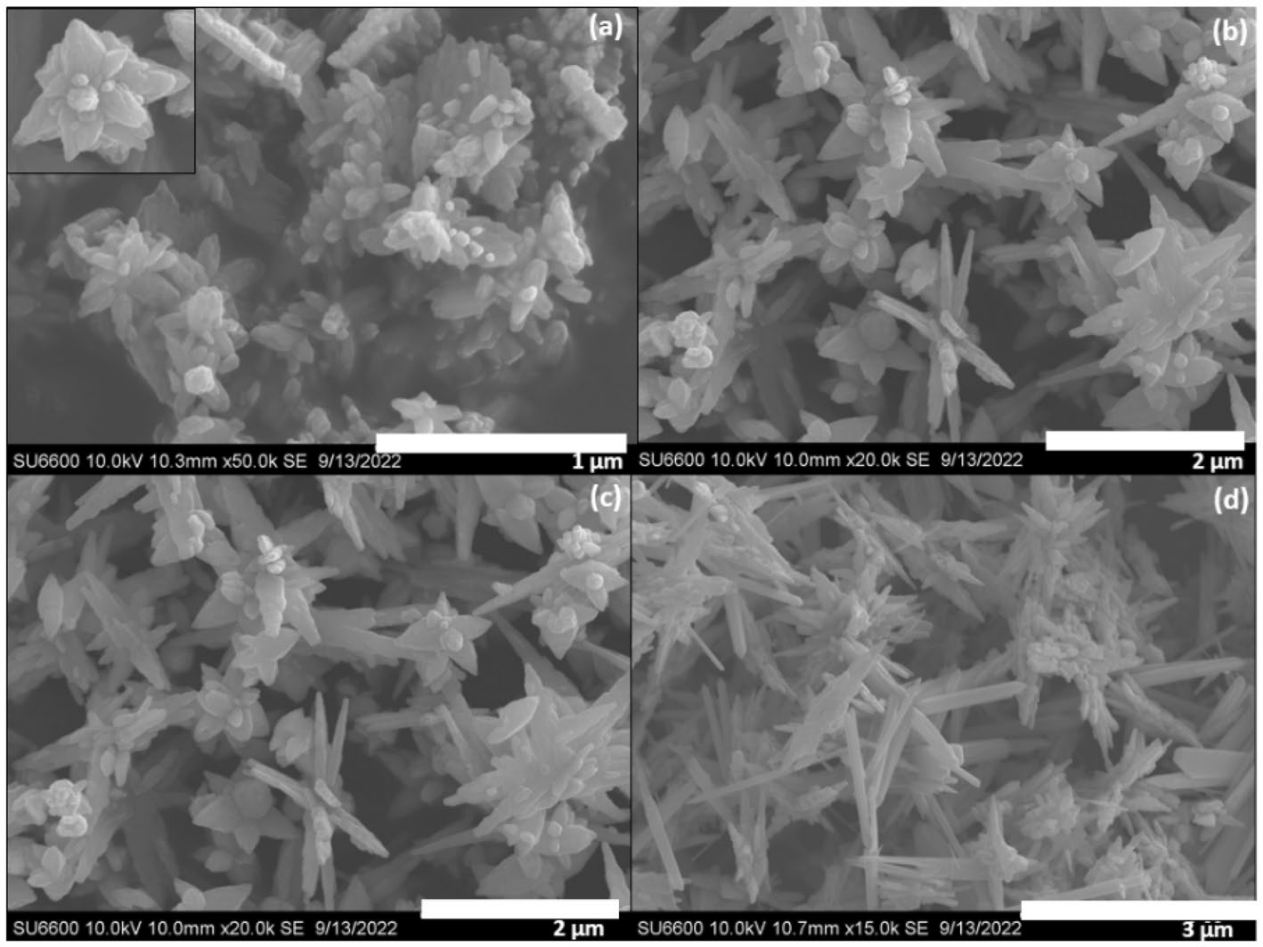
SEM images of the synthesized nanomaterials (**a**) ZnO (**b**) Zn:Cu (2:1) (**c**) Zn:Cu (1:1) (**d**) Zn:Cu (1:2).

SEM images were collected to study the morphology of the synthesized nanomaterials at the macroscale. Flowers-like architectures were observed in the SEM image of pure ZnO (Fig. 2a). Upon addition of NaOH, [Zn(OH^−^)_4_] was formed and further during the hydrothermal treatment ZnO crystal phase was formed. The nucleation and the crystal growth processes control the morphology of the materials where the nucleation occurs followed by the crystal growth. The quantity of the nuclei produced in a weak alkaline solution is rather low, but more crystal growth occurs. Therefore, the ZnO crystal grows along the c-axis around the smaller number of seeds into a petal-like crystal forming a flower-like nanostructure. The width of the petal was about 45 nm.

However, a perfect flower-like structure was not established everywhere and only the petal-like structures were present without organizing around a center ZnO seed. This could be due to the polyethylene glycol polymer co-existing during the formation of the nanostructure which interferes with the proper formation of the flower-like structure due to the bulkiness of the polymer. The morphology of the CuO–ZnO composites clearly shows two different architectures where proper flower-like ZnO are located with CuO rods. As shown in the SEM image of Zn:Cu (2:1) (Fig. 2b) sharp CuO nanorods with a width in the range of 50–230 nm were present with bulky ZnO flower-like architectures. The SEM image of Zn:Cu (1:1) (Fig. 2c) isolated nanorods were not present instead they were aggregated randomly and the images were abundant with CuO nanorods rather than with ZnO flower-like structures. Interestingly, the sharp edges of the rods were not observed and the non-uniform wave-like edges were present. ZnO flower-like structures were completely absent in the SEM image of Zn:Cu (1:2) (Fig. 2d) and are abundant with rods with some rice panicle-like structures. Rods of pencil-like architectures of CuO were present with sharp edges and the rice panicle-like structures of ZnO were present. Rods were relatively abundant compared to corn-like structures as the incorporated Cu is higher in Zn:Cu (1:2).

### TEM analysis

**Figure 3:**
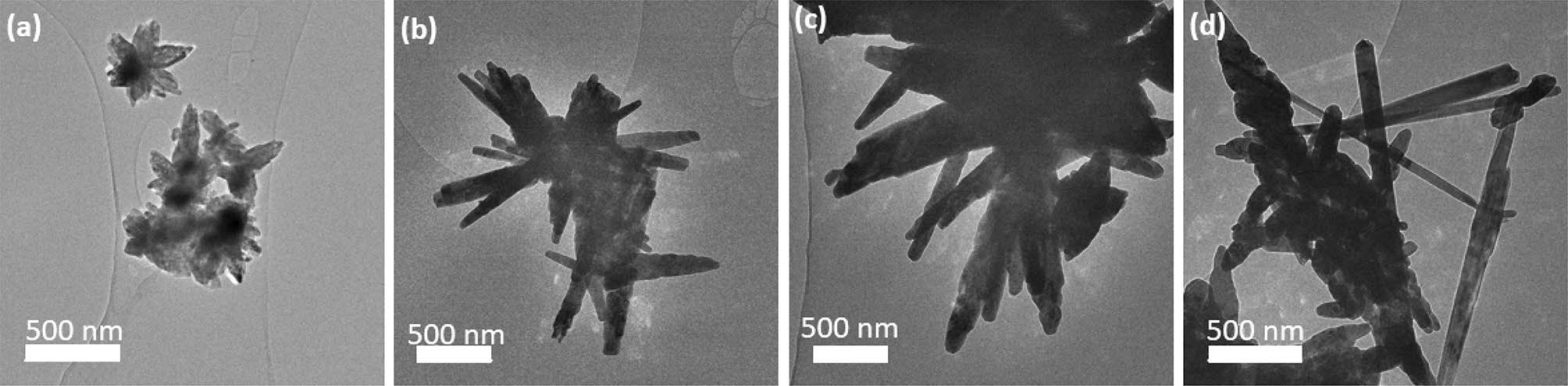
TEM images of the synthesized nanomaterials (**a**) ZnO (**b**) Zn:Cu (2:1) (**c**) Zn:Cu (1:1) (**d**) Zn:Cu (1:2).

TEM images were acquired to further study the morphology of the materials. The 2-D structure of the flower-like arrangement is visible in the TEM image of ZnO (Fig. 3a). However, the features that are more apparent in the 3D structure of the flower-like structures in the SEM images were not prominently present in the TEM image because the detailed structure including the surface roughness and the surface imperfections are more apparent due to the scattering effect of the secondary electrons. The TEM images of Zn:Cu (2:1) and Zn:Cu (1:1) (Fig. 3b,c) show the CuO nanorods and not the ZnO flower-like arrangements. The TEM image of Zn:Cu (1:2) nanocomposite (Fig. 3d) shows the sharp needle-like nanorods with disorganized structures of ZnO deviating from the flower-like arrangement. The supplementary Figs. 2 (a) and (b) of Zn:Cu (2:1) and Zn:Cu (1:1) show the mesoporosity developed in the synthesized nanomaterials due to the biopolymer PEG used in the synthesis.

### XPS analysis

**Figure 4:**
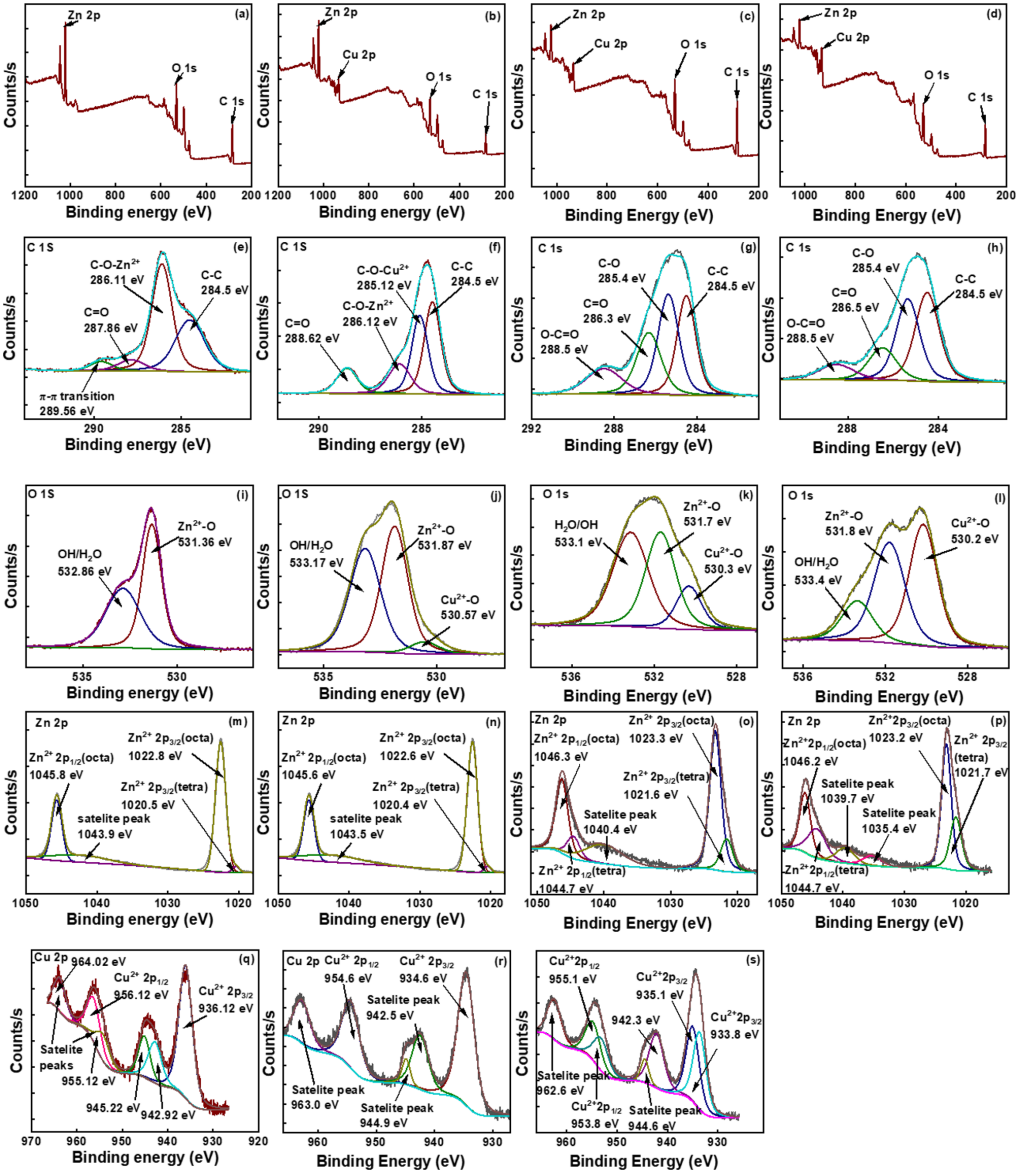
The survey spectra of (**a**) ZnO (**b**) Zn:Cu (2:1), (**c**) Zn:Cu (1:1) (**d**) Zn:Cu (1:2), the higher resolution spectra of C 1 s of (**e**) ZnO (f) Zn:Cu (2:1), (**g**) Zn:Cu (1:1) (**h**) Zn:Cu (1:2), the higher resolution spectra of O 1 s of (**i**) ZnO (**j**) Zn:Cu (2:1), (**k**) Zn:Cu (1:1) (**l**) Zn:Cu (1:2), the higher resolution spectra of Zn 2p of (**m**) ZnO (**n**) Zn:Cu (2:1), (**o**) Zn:Cu (1:1) (**p**) Zn:Cu (1:2), the higher resolution spectra of Cu of (**q**) Zn:Cu (2:1), (**r**) Zn:Cu (1:1) (**s**) Zn:Cu (1:2).

XPS survey spectra of the synthesized nanomaterials were collected to identify the surface elemental composition while the higher resolution spectra were collected to study the surface chemical environments of the individual elements in detail. The survey spectra of ZnO, Zn:Cu (2:1), Zn:Cu (1:1) and Zn:Cu (1:2) are shown in Fig. 4a–d, respectively. The survey spectrum of ZnO shows the presence of Zn, O and C and the survey spectra of the composites exhibit the presence of Cu in addition to those elements. The higher resolution spectra of C 1 s of ZnO shown in Fig. 4e are deconvoluted four peaks at 284.5, 286.11,287.86 and 289.56 eV which are assigned to sp^2^ hybridized C–C, C-O-Zn^2+^, C = O and π-π interactions, respectively. The higher resolution spectra of C 1 s of the nanocomposites Zn:Cu (2:1), Zn:Cu (1:1) and Zn:Cu (1:2) (Fig. 4f–h, respectively show peaks at 284.5, ~ 285.3, ~ 286.3 and ~ 288.5 eV which are attributed to C–C, C-O-Cu^2+^, C-O-Zn^2+^and C = O, respectively. Oxygen in ether bond (C–O–C) of PEG forms dative bonds with both Zn^2+^ and Cu^2+^ creating two different chemical environments in the C-O bond of the composites. The higher resolution spectrum of O 1 s of ZnO (Fig. 4i) is deconvoluted to two peaks at 531.36 and 532.86 eV which are ascribed to Zn^2+^-O and OH/H_2_O, respectively. The higher resolution spectra of O 1 s of the Zn:Cu (2:1), Zn:Cu (1:1) and Zn:Cu (1:2) nanocomposites shown in Fig. 4j–l, respectively, are deconvoluted to three peaks at ~ 530.35, ~ 531.8, ~ 533.2 eV, which are assigned to Cu^2+^-O, Zn^2+^-O and OH/H_2_O, respectively. The ratio between the area under the curve of Zn^2+^-O: Cu^2+^-O of Zn:Cu (2:1), Zn:Cu (1:1) and Zn:Cu (1:2) are 9.6, 2.2 and 0.8, where the area under the curve of Cu^2+^-O dramatically increased with increasing Cu content in the composite. The higher resolution spectrum of Zn 2p of ZnO (Fig. 4m) is deconvoluted to three peaks at 1022.6, 1040, and 1045.6 eV which are attributed to 2p_3/2_ and 2p_1/2_, respectively. The higher resolution spectra of Zn 2p of Zn:Cu (2:1), Zn:Cu (1:1) and Zn:Cu (1:2) are shown in Fig. 4n–p, respectively. Two different chemical environments were observed in both 2p_3/2_ and 2p_1/2_ peaks representing the tetrahedral and octahedral geometries of Zn^2+^ in coordination with oxygen. The higher resolution spectra of Cu 2p of Zn:Cu (2:1), Zn:Cu (1:1) and Zn:Cu (1:2) are exhibited in Fig. 4q–s, respectively. Splitting of Cu 2p_3/2_ and 2p_1/2_ into two sub-peaks as shown in the figures suggests the presence of Cu^2+^ in two different chemical environments which are the Cu^2+^ being complexed with PEG appearing at the low binding energy and the Cu^2+^ appeared at higher binding energy represents the Cu^2+^ present in the lattice.

### Raman spectroscopic analysis

**Figure 5:**
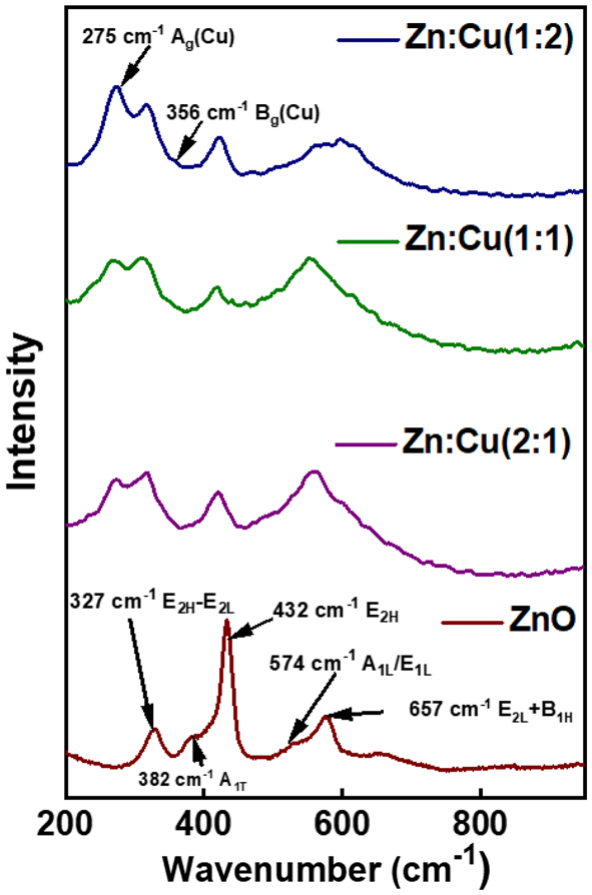
Raman spectra of the synthesized nanomaterials.

The Raman spectra were acquired to confirm the crystallography of the nanomaterials determined by the XRD patterns. The Raman spectra of the synthesized nanomaterials are shown in Fig. 5. The Raman spectrum of ZnO nanomaterial shows Raman bands at 327, 382, 432, 574, and 657 cm^−1^. The basic phonon modes of hexagonal ZnO appeared at 382, 432, and 574 cm^−1^ and are attributed to the A_1T_, E_2H_ and A_1L_/E_1L_, respectively, while the muti phonon scattering modes are represented at 327, and 657 cm^−1^ which are assigned to the E_2H_-E_2L_ and E_2L_ + B_1H_. The Raman spectrum of Zn:Cu (1:2) shows additional Raman bands at 275 and 356 cm^−1^ representing the A_g_ and B_g_ modes of CuO^48,49^. The Raman bands of pure ZnO have been shifted to the low Raman shifts indicating the coupling of ZnO with CuO.

### Antibacterial activity by agar well diffusion method

**Figure 6:**
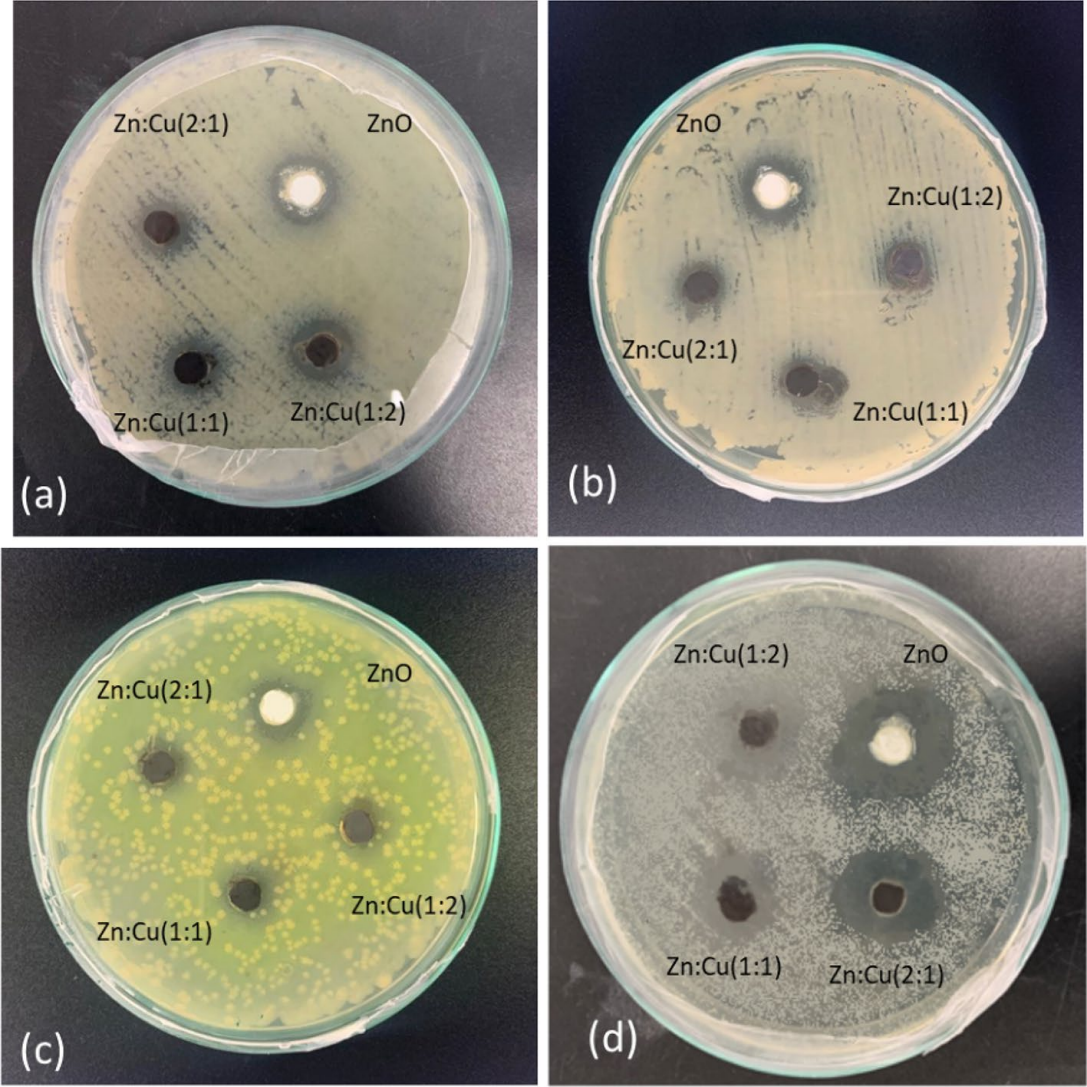
Antibacterial activity of synthesized nano materials (20 mg/ml) with test organisms. (**a**) *E. coli* (**b**) *K. pneumonia* (**c**) *P. aeruginosa* and (**d**) *S. aureus.*

**Figure 7:**
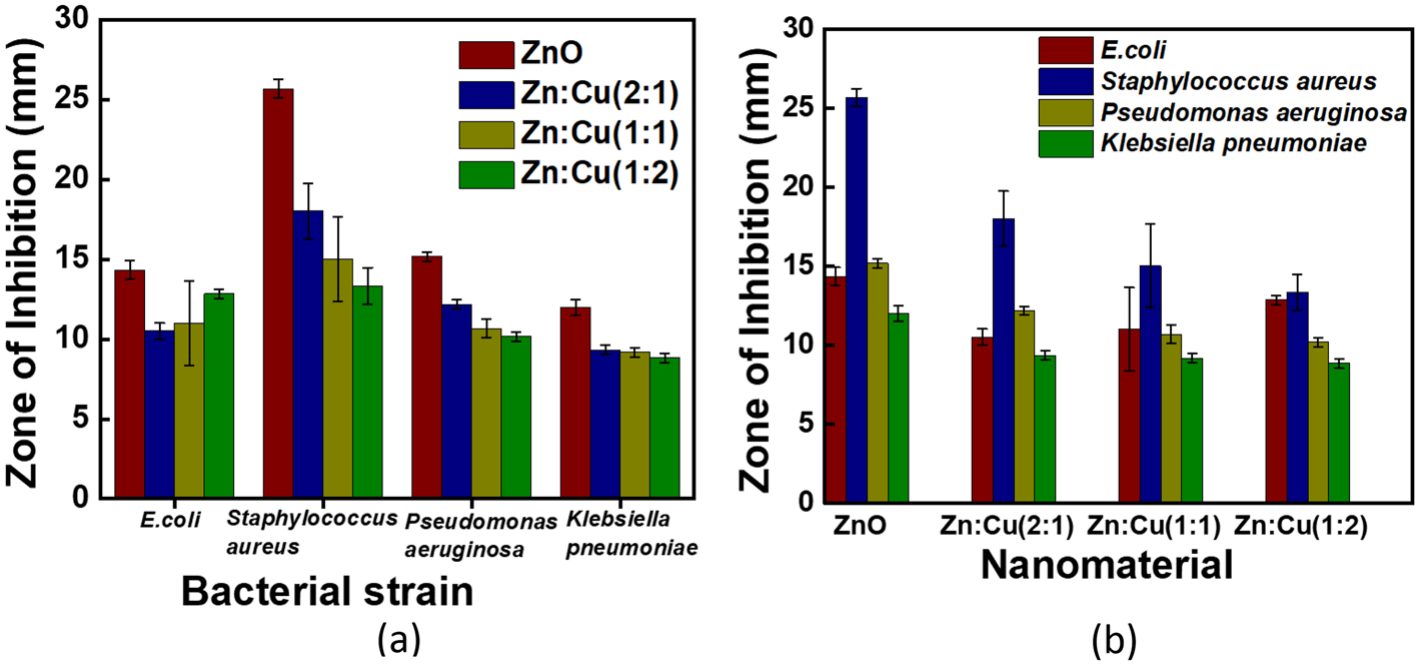
Variation of the zone of inhibition (in mm) against (**a**) the test organisms (**b**) by the synthesized nanomaterials

The metal oxides and metal oxide nanocomposites showed antibacterial activity on both gram-positive and Gram-negative bacterial strains tested. The zone of inhibition of all four samples against the test organisms is shown in Fig. 6. Interestingly the metal oxide nanocomposites were found to be more sensitive towards the Gram-positive strain *Staphylococcus aureus,* than the Gram-negative strains; *Escherichia coli*, *Pseudomonas aeruginosa* and *Klebsiella pneumoniae* as depicted in Fig. 7a. For example, the diameter of the zone of inhibition for *Staphylococcus aureus* by ZnO nanomaterials is 25.67 ± 0.58 mm while the same for *Escherichia coli*, *Pseudomonas aeruginosa* and *Klebsiella pneumonia* are determined to be 14.33 ± 0.58 mm, 15.17 ± 0.29 mm and 12.00 ± 0.5 mm, respectively. Among the composite nanomaterials Zn:Cu 2:1 showed the highest antibacterial activity for *Staphylococcus aureus* and *Pseudomonas aeruginosa* with diameters of 18.00 ± 1.73 mm and 12.17 ± 0.29 mm, respectively, while the antibacterial activity of all the composites were quite similar on *Klebsiella pneumonia* with an average diameter of 9.11 ± 0.25 mm. A different behaviour was observed for *Escherichia coli* where the highest antibacterial activity among the composite nanomaterials was obtained in the presence of Zn:Cu 1:2 (12.83 ± 0.29 mm) and the least was found to be with Zn:Cu 2:1 (10.50 ± 0.50 mm). The zone of inhibition and hence the antibacterial activity of the nanomaterials decrease with a decreasing proportion of Zn^2+^ for *Staphylococcus aureus*, *Pseudomonas aeruginosa* and *Klebsiella pneumonia*. However, such a trend was not observed for *Escherichia coli*. Figure 7b shows the zone of inhibition produced by different metal oxide nanoparticles against both Gram-positive and Gram-negative bacterial strains.

The effect of the concentration of the nanocomposite on the inhibition of the bacteria was further investigated varying the concentration as 20, 40 and 60 mg/ml. The diameters of the inhibition zones are tabulated in Table 2. The inhibitory action of the nanocomposites on the growth of *E.Coli* increased with increasing concentration in all the composites. For example, the diameter of the inhibition zone increased from 10.50 ± 0.50 mm with 20 mg/ml to 12.83 ± 0.29 mm with 40 mg/ml and to 13.50 ± 0.50 mm with 60 mg/ml when Zn:Cu 2:1 is used as the nanocomposite. A similar trend was observed with all the composites. In general, all the antibacterial mechanisms applicable and will be discussed in section "Antibacterial activity by agar well diffusion method" have increasingly affected the bacteria with increasing doses of the antibacterial reagent which is the nanocomposite. The same antibacterial behaviors were observed for *Pseudomonas aeruginosa* and *Klebsiella pneumonia* where only the inhibitory action decreased with increasing concentration of *Pseudomonas aeruginosa* with ZnO nanomaterial. A different behaviour was observed for *Staphylococcus aureus* where the antibacterial activity of all the nanocomposites decreased moving from 20 to 40 mg/ml and again increased when the concentration was increased to 60 mg/ml. The diffusion of the nanomaterials from the well to the medium has lowered when the concentration of the nanocomposite increased from 20 mg/ml to 40 mg/ml lowering the physical damage caused to the bacterial species and hence resulting in lower antibacterial activity. However, when the concentration is increased from 40 mg/ml to 60 mg/ml, though the physical damage caused by the nanomaterials through the restriction of nanoparticle diffusion lowers the antibacterial activity, other antibacterial mechanisms profoundly become active and more prominent and cause an increase in the antibacterial activity on *Staphylococcus aureus*.

**Table 2:** Variation of the mean diameter of the inhibition zone with increasing concentration of the nanocomposites.

Test organism	ZnO			Zn:Cu 2:1			Zn:Cu 1:2			Zn:Cu 1:1		
	20 mg/ml	40 mg/ml	60 mg/ml	20 mg/ml	40 mg/ml	60 mg/ml	20 mg/ml	40 mg/ml	60 mg/ml	20 mg/ml	40 mg/ml	60 mg/ml
*E. Coli*	14.33 ± 0.58	14.83 ± 2.02	15.17 ± 0.76	10.50 ± 0.50	12.83 ± 0.29	13.50 ± 0.50	11.00 ± 2.65	12.67 ± 0.76	12.83 ± 0.29	12.83 ± 0.29	13.58 ± 1.38	13.00 ± 0.29
*Staphylococcus aureus*	25.67 ± 0.58	17.75 ± 0.66	20.00 ± 1.15	18.00 ± 1.73	14.17 ± 1.44	16.17 ± 1.61	15.00 ± 2.65	12.33 ± 0.58	14.17 ± 1.53	13.33 ± 1.15	12.50 ± 0.00	14.33 ± 0.76
*Pseudomonas aeruginosa*	15.17 ± 0.29	13.67 ± 0.29	12.50 ± 0.00	12.17 ± 0.29	12.83 ± 2.02	13.08 ± 0.14	10.67 ± 0.58	12.33 ± 0.58	12.67 ± 0.29	10.17 ± 0.29	12.33 ± 0.29	14.08 ± 1.81
*Klebsiella pneumoniae*	12.00 ± 0.50	14.17 ± 2.25	15.50 ± 0.50	9.33 ± 0.29	10.83 ± 0.76	11.00 ± 0.50	9.17 ± 0.29	10.25 ± 0.90	11.67 ± 0.29	8.83 ± 0.29	11.17 ± 1.26	11.50 ± 0.87

### Time-kill synergy assay

**Figure 8:**
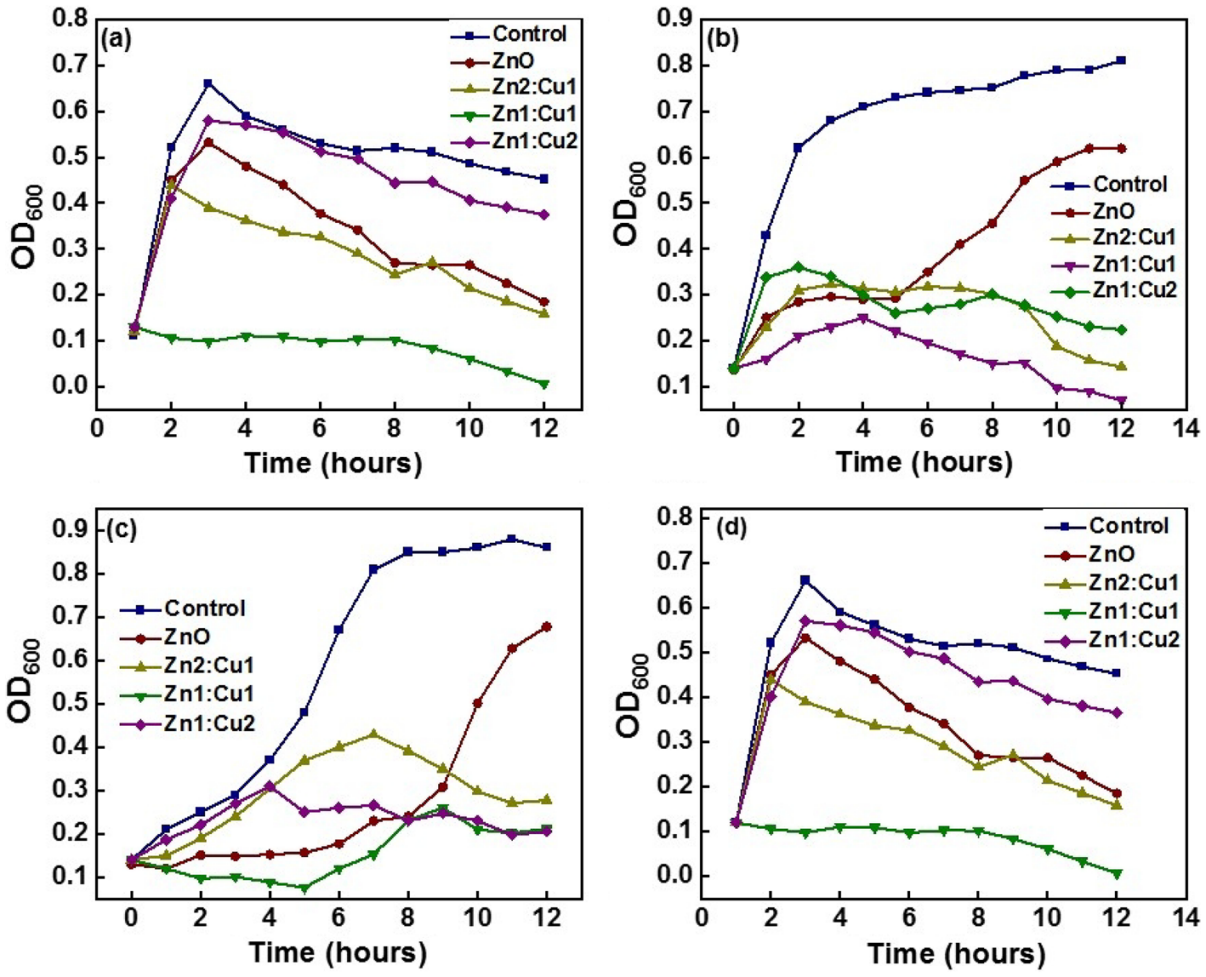
Time-kill curves of the test organisms (**a**) *Escherichia coli* (**b**) *Klebsiella pneumoniae* (**c**) *Pseudomonas aeruginosa* and (**d**) *Staphylococcus aureus* against the synthesized nanomaterials.

The antibacterial activity of the composites was then evaluated in the liquid medium through the determination of the time-kill synergy assay of the bacteria tested. In-vitro bacterial growth is inhibited by ZnO and CuO nanomaterials in similar studies^7,25,27,43,47,50–54^. The concentration of the nanomaterials (20 mg/ml) demonstrated significant antibacterial action against gram negative *Escherichia coli*, *Pseudomonas aeruginosa* and *Klebsiella pneumoniae*. Gram positive *Staphylococcus aureus* was similarly suppressed at concentrations of 20 mg/ml. Figure 8 displays the time-kill curves obtained from the tested bacterial pathogens against all of the investigated bacterial nanomaterials. Bacterial suspensions in LB broth were used in a time-kill kinetic test for 12 h with the addition of nanomaterials (20 mg/ml) and the observations were taken at 600 nm.

### MIC and MBC assay

The MIC and MBC values were determined for all four test microorganisms. MIC is defined as the lowest concentration of a material that can inhibit the visible growth of an organism; whereas MBC is defined as the lowest concentration of a material that inhibits the growth of an organism in batch cultures, this can be determined from broth dilution MIC tests by subculturing to agar media without antibiotics^7,47,51^. The obtained MIC, MBC, and MBC/MIC values are shown in Table 3.

**Table 3:** MIC, MBC and MBC/MIC values of the nanomaterials against the test organisms.

Bacterial Strains	MICs (mg/mL)				MBCs (mg/mL)				MBC/MIC values			
	ZnO	Zn:Cu 2:1	Zn:Cu 1:1	Zn:Cu 1:2	ZnO	Zn:Cu 2:1	Zn:Cu 1:1	Zn:Cu 1:2	ZnO	Zn:Cu 2:1	Zn:Cu 1:1	Zn:Cu 1:2
*E. coli*	0.313	0.313	0.625	0.625	2.500	0.313	5.000	5.000	8	1	8	8
*S. aureus*	0.313	5.000	5.000	2.500	1.250	10.000	10.000	10	4	2	2	4
*K. pneumoniae*	2.500	2.500	20.000	40.000	5.000	5.000	10.000	10.000	2	2	0.5	0.25
*P. aerugenosa*	0.313	20.000	0.313	5.000	20.000	20.000	2.500	2.500	64	1	8	0.5

The antibacterial activity was further evaluated based on the MBC/MIC ratio. If the MBC/MIC ratio ≤ 4, the effect is bactericidal and if the MBC/MIC > 4, the effect is bacteriostatic^55^. The MBC/MIC ratios are shown in Table 3. The MBC/MIC ratio for *E. coli* in the presence of ZnO, Zn:Cu 1:1 and Zn:Cu 1:2 were greater than 4 suggesting that they cause bacteriostatic effect while Zn:Cu 1:2 showed the bactericidal effect on the growth of *E. coli*. Similarly, ZnO and Zn:Cu 1:1 were bacteriostatic against *Pseudomonas aeruginosa* while Zn:Cu 2:1 and Zn:Cu 1:2 exhibited bactericidal effect*.* All the nanomaterials showed bactericidal effect on both *Staphylococcus aureus* and *Klebsiella pneumoniae*. Interestingly, Zn:Cu 2:1 showed the bactericidal effect on all the bacterial strains tested.

#### PEG functionalization

In this study, the antibacterial activity in terms of zone of inhibition of PEG-coated ZnO nanomaterials for *Escherichia coli****,**** Pseudomonas aeruginosa*, *Klebsiella pneumoniae* and *Staphylococcus aureus* (14.33 ± 0.58, 15.17 ± 0.29, 12.00 ± 0.50 and 25.67 ± 0.58 mm, respectively,) was found to be significantly higher than the naked ZnO nanomaterials (10.83 ± 1.04, 13.00 ± 2.60, 10.83 ± 1.15 and 10.33 ± 1.89 mm, respectively,) confirming the capability to enhance the biocompatibility of the fabricated nanomaterials against tested microorganisms.

## Discussion

### Antibacterial activity by agar well diffusion method

The agar well diffusion method was performed to evaluate the antibacterial activity of the synthesized nanocomposites. Based on prior research findings and the preliminary trials conducted, it was found that the inhibition zone is greater in the well-diffusion method than in other assays like disk diffusion, hence the agar well-diffusion test was used for this study. This is so that the well-diffusion test may increase the antibacterial activity by doubling the volume of nanomaterial suspensions and increasing nanomaterial diffusion through the medium^25,56^. To our knowledge, no research has been published about the antibacterial activity of the ZnO and CuO nanomaterials synthesized with PEG functionalization in the ratios and co-precipitation method used in this study. Nevertheless, numerous publications have cited that metal nanomaterials show antibacterial activity and that PEG functionalization improves the bactericidal effect of nanomaterials^25,51,56–59^.

**Figure 9:**
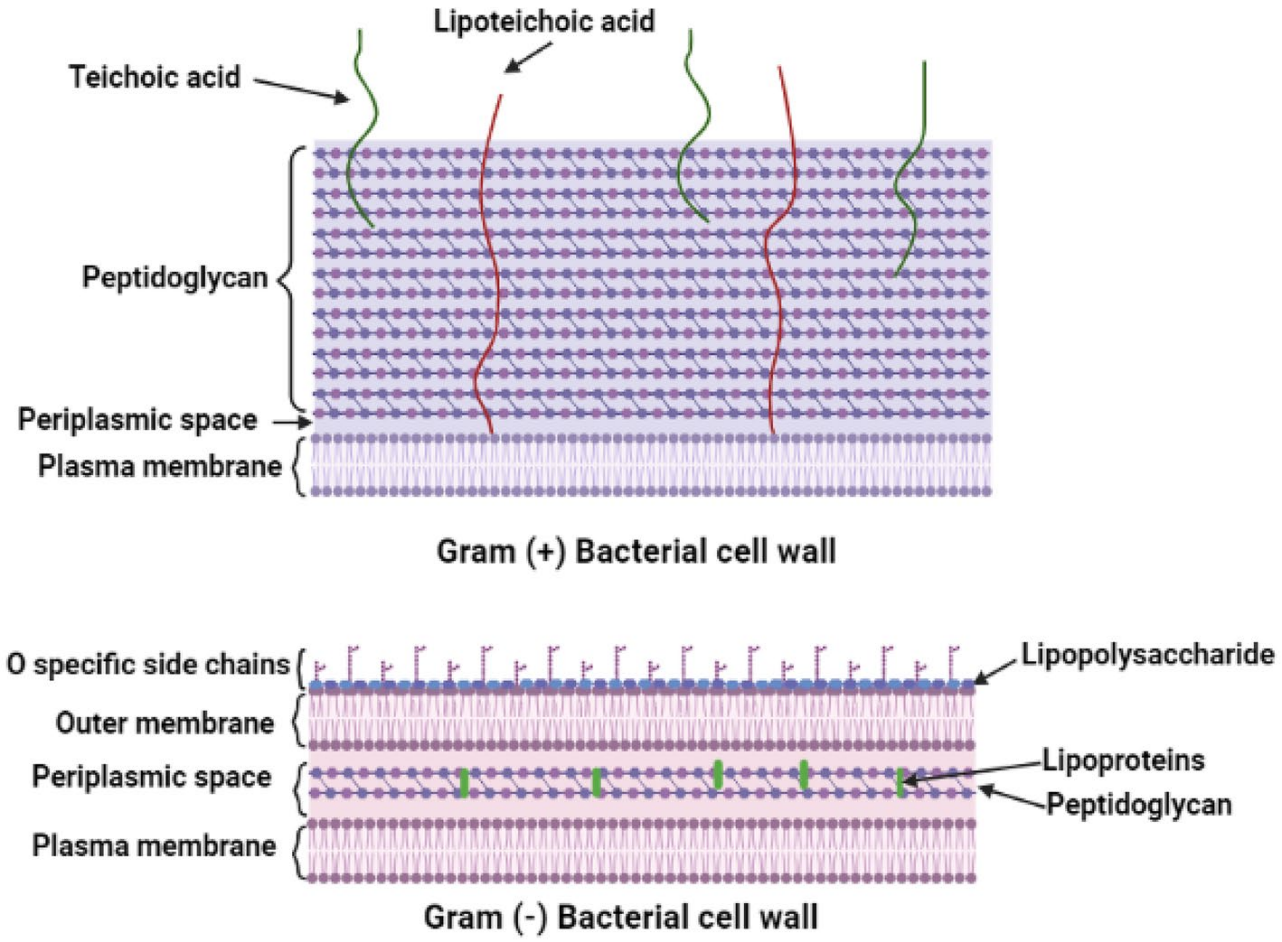
The cell wall structure of Gram-positive and Gram-negative bacteria.

Depending on the type of microorganism, different metal oxide nanoparticles have different levels of microbial sensitivity. Understanding the distinctions between the cell walls of Gram-positive and Gram-negative bacteria is crucial since the primary toxicological effect that antimicrobial substances exert on bacteria occurs when they come into direct contact with the cell surface^28^. The structure of the Gram positive and Gram negative cell walls is illustrated in Fig. 9 The surface of bacteria, both Gram-positive and Gram-negative, is negatively charged^27^. The peptidoglycan layer of gram-positive bacteria is composed of linear chains that alternate N-acetylglucosamine (NAG) and N-acetylmuramic acid (NAM) residues. These chains are linked together by an arrangement of 3 to 5 amino acids that cross-link one another to form a cohesive mesh. Most Gram-positive bacteria also have negatively charged teichoic acids (with substantial phosphate groups) that stretch from the cell wall to the surface. On the other hand, gram-negative bacteria have a significantly more complex structure. Gram-negative bacteria have an outer membrane made of phospholipids and partly phosphorylated lipopolysaccharides (LPS), in addition to the thin layer of peptidoglycan, which helps to enhance the negative surface charge of their cell envelope^60^. Electrostatic interactions cause positively charged nanoparticles to be attracted to the surface of negatively charged bacterial cell walls. Positively charged metal-based nanoparticles, on the other hand, form a firm bond with membranes, disrupting cell walls and therefore increasing permeability^61^.

**Figure 10:**
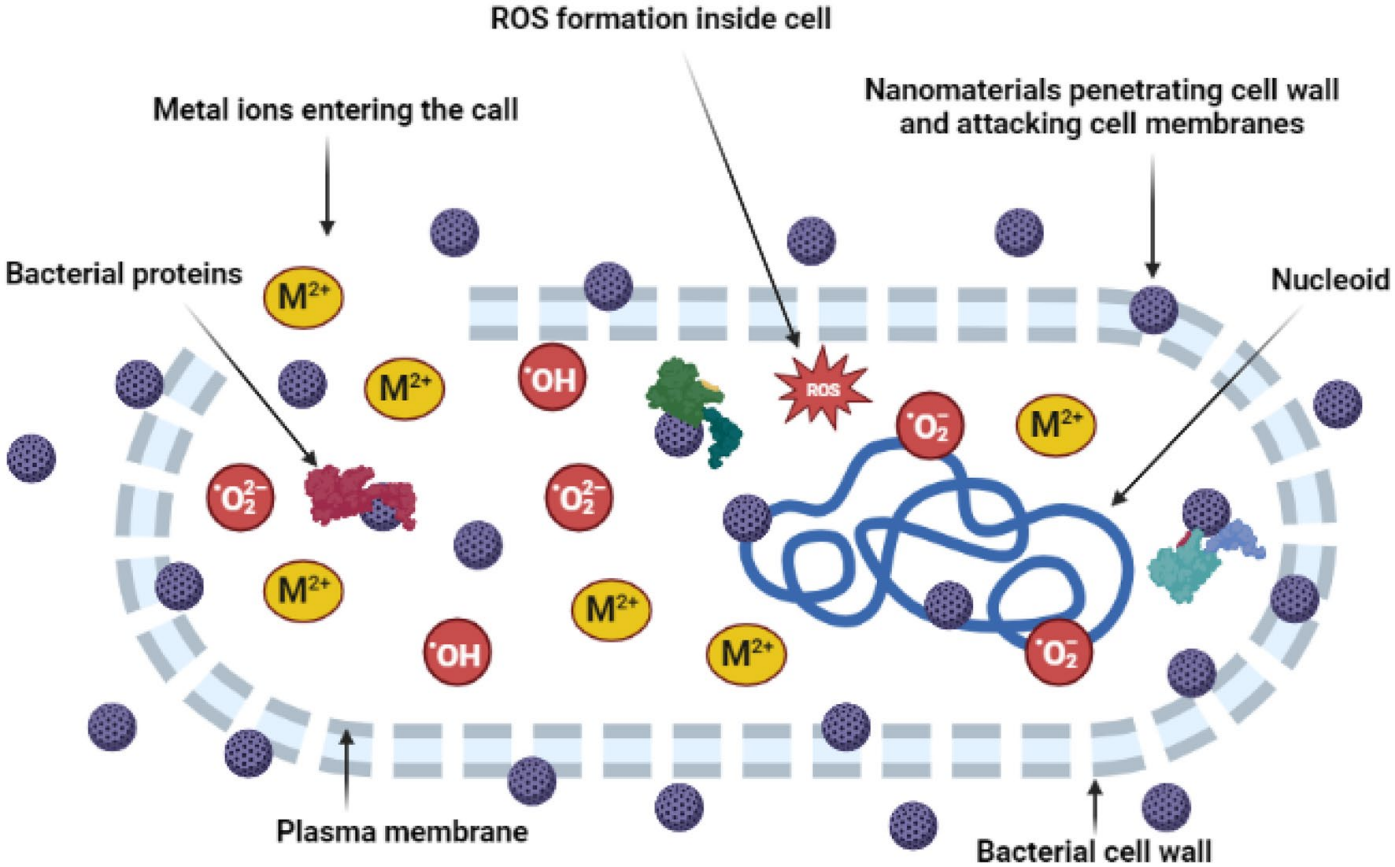
Nanoparticles, ROS and metal ions damage the bacterial cell wall, cell membrane, proteins and nucleic acid.

Additionally, metal ions released by nanoparticles from the extracellular environment can penetrate cells and interfere with biological processes^61^. When the metal ions are free to interact with biological components like proteins, membranes, and DNA, cell functions are disrupted^61^. Reactive oxygen species (ROS) can be produced inside the cell by metal ions or nanoparticles^62^. A variety of ROS can be formed by nanomaterials in cells, i.e.,·O_2_^−^, ^1^O_2_, OH, and H_2_O_2_ which can take part in physiological and pathological cellular processes^62–64^. The cells have a variety of repairing and antioxidant mechanisms for defence and tripeptide glutathione is one of the most effective antioxidants^62^. Upon exposure to ROS glutathione is oxidized as a result of the oxidative stress it causes, and bacteria's antioxidant defence system against ROS is suppressed. Thus, the glutathione depletion may be a defining sign of the negative effects imposed on by nanomaterials' prooxidative actions in cells^62,65^. Hence the overall antibacterial effect will be caused by the metal nanomaterial, the ROs generated, and the metal ions as shown in Fig. 10.

**Figure 11:**
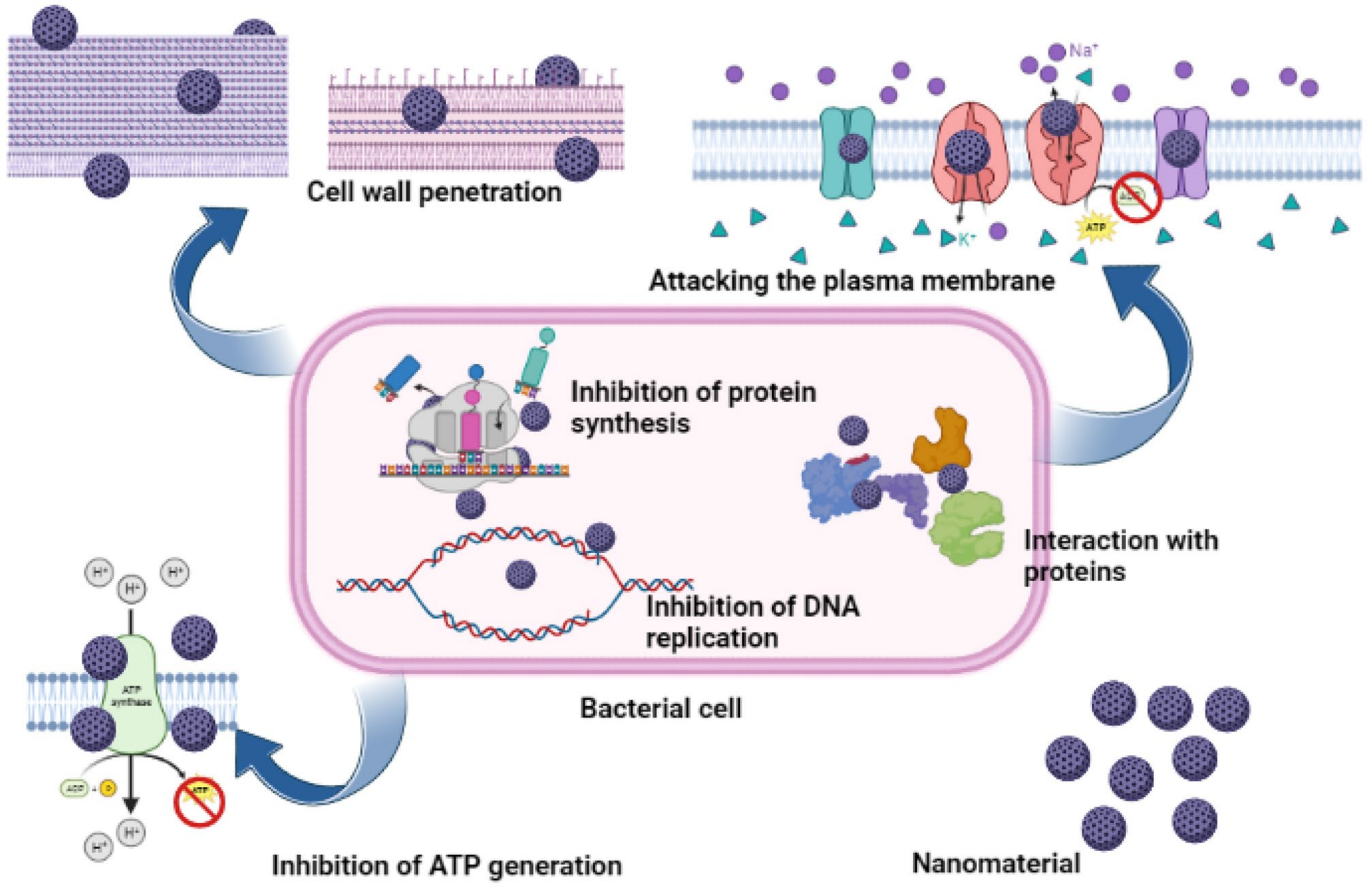
Schematic illustration of interactions between nanomaterials and bacterial cells.

Furthermore, it was found that pure ZnO nanomaterials show superior antibacterial activity against the four bacterial species tested compared to the composites synthesized as shown in Fig. 7b while the nanoparticles are located in the wells, the bacteria are inoculated on the surface of the MHA media. Therefore, either the nanoparticles or the metal ions of the nanoparticles should diffuse through the solid agar medium to interact and inhibit the growth of bacteria. It is difficult to assume that the flower-like arrangement of ZnO and CuO rods is diffusing through a solid medium due to the size and corresponding steric hindrance. Hence, it should be the metal ions, Zn^2+^ and Cu^2+^ which should easily diffuse through the medium. Strong coordination bonds can be formed between metal ions with the N, O, or S atoms that are prevalent in organic molecules and biomolecules. These biomolecules' functioning can be impacted by the binding of metal ions with them. Metal ion-associated antibacterial medicines frequently exhibit broad-range activity since the binding relationship between metal ions and biomolecules is typically nonspecific^30^. Metal ions disrupt the cell wall and the cytoplasmic membrane and once they enter the bacterial cell, they denature the ribosomes and interfere with the protein synthesis. Further, the metal ions would interrupt ATP production because metal ions deactivate respiratory enzymes on the cytoplasmic membrane. By altering the charge balance of bacteria, zinc ions can cause them to undergo apoptosis^66^ (Fig. 11).

Bacterial cells are affected by Cu^2+^ ions leading to microbial growth inhibition. Recently, the use of Cu nanoparticles or CuO nanoparticles as antibacterial agents has been investigated^67,68^. Recently Cu nanoparticles have been incorporated into polymers such as starch hydrogels and functional polymer coatings which would show broad-spectrum antimicrobial activity^69,70^. Both ROS generation and release of Cu ions from Cu nanoparticles are considered to be responsible for contact killing of bacteria, and it is presumed that Cu nanoparticles-incorporated materials are capable of eradicating both Gram-positive and Gram-negative bacteria through the "attract-kill-release" route. Additionally, it has been discovered that Cu nanoparticles-incorporated coatings can also inhibit *E. coli* biofilm formation on the surfaces, which is important for the prevention of infections. Although CuO nanoparticles have been shown to have antibacterial activity, the stability of Cu nanoparticles in the ambient air and their rapid oxidation could pose a challenge to their practical usage^30^. This could be the possible reason for the reduced zone of inhibitions given by Cu-incorporated nanocomposites compared to ZnO alone.

Additionally, Zn^2+^ ions play an important part in many physiological functions, yet at certain levels, they are harmful to cells. Releasing of zinc ions in media comprising ZnO nanoparticles and bacterial cells is one of the key antibacterial mechanisms for ZnO nanoparticles^18,26,71–73^. It appears that different cellular targets harbouring a variety of reactions and responses are related to the mode of antibacterial action of Zn^2+^ ions^74,75^. Zn^2+^ is known as a sulfhydryl reactive agent, which can form bonds with sulfhydryls (–SH) in cells which causes inhibition of growth in cells and cell death^76^. It has been discovered that the metal oxide nanomaterial complex ions get released in an aqueous medium and it depends on both the dissolving and adsorption processes of the nanomaterial. While the CuO nanomaterial antibacterial impact arises from both the released Cu^2+^ and the CuO particles, the ZnO nanomaterial’s antibacterial effect is mostly due to the released Zn^2+^^7^. The dissolution rate of ZnO nanoparticles is significantly higher than the other CuO nanoparticles^77,78^. In a similar study, it was shown that 1–4.5 mg/L of Zn^2+^ were detected at the ZnO concentration of 5.0 mg/L, which was close to the reported aqueous solubility of ZnO (1.6–5 mg/L)^78^. Additionally, it was discovered that over time, the amount of dissolved Zn^2+^ in ZnO suspensions increased which was similar to how the bactericidal effects of both ZnO suspensions and ZnO supernatants changed, indicating a link between the Zn^2+^ release and the antibacterial properties of ZnO nanoparticles^78,79^. Because the dissolution of ZnO is greater than that of CuO and hence, the concentration of Zn^2+^ diffuse through the agar medium would be higher than that of Cu^2+.^ Moreover, the amount of Zn^2+^ diffused from a given weight of the nanomaterial in the composites would be lesser than that from the pure ZnO. Hence, the antibacterial activity of pure ZnO has resulted to be greater than that of CuO. However, it should also be noted that Gram-positive bacterial strain, *Staphylococcus aureus* had the highest inhibition-zones, 44%, 41% and 53% higher than Gram-negative bacterial strains *Escherichia coli*, *Pseudomonas aeruginosa* and *Klebsiella pneumoniae*, respectively, in the case of ZnO nanoparticles. This observation suggests a higher Gram-negative strain resistance/tolerance against the ZnO functionalized with PEG, over Gram-positive bacterial strains. This finding is in agreement with similar studies which reported that the ZnO nanoparticle effect is more pronounced against Gram-positive bacterial strains than Gram-negative bacterial strains^7,80^.

### Time-kill synergy assay

The time-kill curve of nanomaterials against all of the studied bacterial pathogen strains demonstrated time-dependent rapid bactericidal action, which directly affected the bacterial cells before they reached the stationary phase. The growth curves of bacteria exposed to nanomaterials show that they can inhibit both bacterial growth and reproduction. We have demonstrated that synthesized nanomaterials can inhibit the growth of Gram-negative and Gram-positive bacteria using MIC tests, MBC tests and conventional growth curves. All of the investigated bacterial development was proven to be blocked or slowed down by Cu:Zn 1:1 nanomaterial during the lag phase and log phase. There is a rapid rise in cellular metabolism during the lag phase leading to the active production of cellular macromolecules, primarily enzymes^81^. Cells begin consistently dividing during the log (exponential) phase via the binary fission process. The culture grows at the highest rate possible, exponentially. In other words, the increase in the number of cells is proportional to the size of the current population:

$$dn{/}dt = \alpha n$$

where n is the number of bacteria present in the culture medium at time t and *α* is a constant known as the "specific growth rate." Integration yields the well-known logarithmic growth relation:

$$\begin{aligned} & \ln ({\text{n}}/{\text{n}}_{0} = \alpha \left( {{\text{t}} - {\text{t}}0} \right){\text{or}} \\ & {\text{n}} = {\text{n}}_{0} {\text{e}}^{{\alpha ({\text{t }} - {\text{t}}0)}} \\ \end{aligned}$$

where n_0_ is the initial number of bacteria at a time t_0_ when the lag phase concludes or the log phase begins, and n_0_ is the initial number of bacteria at that moment.

Thus, below is the formula for the generation time, often known as the "doubling time," (τ) of the cell population^82^.

$$\tau = \ln 2{/}\alpha$$

Because the specific growth rate α, and the number of bacterial cells at the point of completion in lag phase n, change due to the bactericidal effect of metal oxides nanocomposites, "doubling time," (τ) may also be changed in the media where synthesized nanomaterials are introduced in comparison with the non-treated control. Moreover, distinct phases of a typical bacterial growth curve have also been affected by the contact of nanomaterials synthesized. Ultimately a bacterial cell size that is appropriate for a particular environmental situation and the developmental fate is achieved by the coordination of cell growth and division^83^. Once that coordination is disrupted the developmental fate is influenced. These findings suggest that synthesized nanomaterials are highly efficient in the lag and log phases against all of the investigated bacterial pathogens.

The Zn:Cu (1:1) sample showed the highest antibacterial activity against all the bacterial species tested being different to ZnO which showed the highest antibacterial activity in the agar well diffusion method. CuO needle-like nanorods perforate the cell wall and cell membrane and get into the bacterial cell easily due to the size and the shape of the nanomaterial in which the diameter of the nanorods is in the range of 50–230 nm where they can easily pass through the channels of which the diameter is in the micrometer level. Further, flower-like ZnO materials also cause physical damage to the bacterial cells. However, penetration of such nanostructures into the cells is not feasible due to the steric hindrance of them. The antibacterial mechanisms of the nanocomposites in the liquid medium are mainly contributed by the metal ions effect and the damage caused by the CuO nanorods. Metal nanopales due to their small particle size and large surface area have contributed to increasing their antibacterial action and cause cytotoxicity in bacteria^84^. The metal ions Zn^2+^ and Cu^2+^ cause inhibitory effects on the bacteria via different mechanisms as described above.

The contribution from both effects is equally effective when Zn:Cu (1:1) is used as the nanomaterial giving the maximum antibacterial effect. The other composites, Zn:Cu (2:1) and Zn:Cu (1:2) are less effective than the Zn:Cu (1:1) because the contribution from one effect is lesser than the other in both circumstances. The effect of metal ions especially of Zn^2+^ is greater when Zn:Cu (2:1) is used and the effect of CuO nanorods is higher once Zn:Cu (1:2) is used as the antibacterial agent. Pure ZnO is contributed mainly by the release of the Zn^2+^ and the physical damage caused to the bacterial cells by the collisions of ZnO with bacterial cells may have also contributed. Since Zn^2+^ can only pose the bacteriostatic effect on microorganisms it is evident that the ZnO flower-like nanomaterials and the ROs generated from Zn nanomaterials have also contributed to the overall inhibition of bacteria^74^. Aquatic ZnO-nanomaterials suspensions have been reported to result in an increased level of ROS. ROS production has been identified as one of the primary sources of nanotoxicity in many studies^85–88^. The antibacterial activity has been attributed to the release of ROS onto the surface of ZnO-nanomaterials under UV and visible light, and the ROS release resulted in the death of bacteria. The researchers presented the following explanation for the generation of ROS (OH-, H_2_O_2_, and O_2_^2−^) on the ZnO surface and suggested a relationship between photon reactions and antibacterial activity. Water (H_2_O) and the electron and hole interact to create ·OH and H^+^. Additionally, superoxide anion (·O_2_^−^), produced by O_2_ molecules (suspended within the combination of bacteria and ZnO), interacts with H^+^ to create HO·_2_ which interferes with electrons to produce hydrogen peroxide (·HO_2_), which then reacts with H^+^ to produce molecules of hydrogen peroxide (H_2_O_2_). The latter can travel across membranes and kill or injure bacteria there. The surface of ZnO nanomaterials plays a major role in the generation of H_2_O_2_ by producing extra-active molecules which can harm bacterial cells^89^.

$${\text{ZnO}} + {\text{h}}\nu \to {\text{e}}^{ - } + {\text{H}}^{ + }$$

$${\text{H}} + {\text{H}}_{2} {\text{O}} \to ^{ \cdot } {\text{OH}} + {\text{H}}^{ + }$$

$${\text{e}}^{ - } + {\text{O2}} \to^{ \cdot } {\text{O}}_{{2}}^{ - }$$

$$^{ \cdot } {\text{O}}_{2} + {\text{H}}^{ + } \to {\text{HO}}_{2}^{ \cdot }$$

$${\text{HO}}_{2}^{ \cdot } {\text{ + H}}^{ + } + {\text{e}}^{ - } {\text{H}}_{{2}} {\text{O}}_{{2}}$$

It is widely known that copper causes DNA damage and ROS production through Fenton-like and other processes. CuO nanoparticles' strong antibacterial action is caused by the ROS that are produced by the nanoparticles attached to the bacterial cells, which in response causes an increase in intracellular oxidative stress.

$${\text{Cu}}^{ + } {\text{ + H}}_{{2}} {\text{O}}_{{2}} \to {\text{Cu}}^{{{2} + }} + {\text{HO}}^{ - } + {\text{HO}}^{ \cdot }$$

### MIC MBC assay

The gram-positive bacteria *Staphylococcus aureus* as well as the gram-negative bacteria *Escherichia coli, Pseudomonas aeruginosa* and *Klebsiella pneumoniae* showed promising antibacterial activity. Despite the prevalent belief that bactericidal antibacterial agents are more effective than static antibacterial agents, this is not supported by adequate literature. The terms "cidal" and "static" are both used to describe the effects of antibacterial drug concentrations on bacterial growth over a specific tolerance. Antibacterial drugs that target bacterial protein synthesis are mostly bacteriostatic while those that target bacterial cell walls are generally bactericidal^90^. For instance, bactericidal drugs like cephalosporins and other beta-lactam antibiotics hinder or impede the formation of bacterial cell walls. In contrast, bacteriostatic antibiotics like chloramphenicol and clindamycin function by preventing or slowing down bacterial growth by blocking protein synthesis. Fundamental data on an antibacterial agent's me chanism of action is provided by the MIC and MBC assays^43,45,91^.

An antibacterial agent’s minimum bactericidal concentration (MBC) is its lowest concentration of bactericidal activity. It is found by re-culturing (subculturing) broth dilutions (i.e., those at or above the MIC) that prevent the development of a bacterial organism^92^. After streaking the broth dilutions onto agar, they are left to incubate for 24 h to 48 h. The MBC is the lowest antimicrobial broth dilution that prevents the organism from growing on the agar plate. The organism's inability to proliferate on the plate suggests that there are only nonviable organisms there. Previous research conducted by Kotb et al. has also demonstrated that silver nanoparticles against methicillin-resistance (MRSA) and methicillin-susceptible Staphylococcus aureus (MSSA) strains have equal MBC and MIC values indicating the overall bactericidal effect towards the microorganisms similar to that of Cu:Zn 1:2 against *E. coli* is bactericidal^93^. This may occur if the lowest concentration of a material that can inhibit the visible growth of an organism is the same as the lowest concentration of a material which can inhibit the growth after subsequent culturing.

The antibacterial activity of copper, cobalt, silver and zinc has been studied via MIC and MBC assays against *S. aureus*, *E. coli* and *S. epidermidi* by *Farah *et al*.* Compared to copper and cobalt nanoparticles, zinc and silver have shown stronger antibacterial activity. *S. aureus* was found to have a greater sensitivity against zinc and silver than *E. coli*, although both bacteria have shown comparable sensitivity patterns against copper and cobalt nanoparticles. *S. aureus* displayed a greater MIC for copper in comparison to silver and zinc, indicating increased efficacy of zinc and silver as well as strain specificity. *E. coli*, on the other hand, displayed equal MICs for copper, zinc, and silver nanoparticles, except for cobalt^41^.

### PEG functionalization

Antibacterial activity is increased, utilizing polyethyleneglycol (PEG) as a surface functionalization agent. Studies have also demonstrated the stability of PEG functionalized nanomaterials following administering in vitro and in vivo settings^94^. Additionally, these were discovered to be more successful at killing cells by penetrating the cell membrane^95,96^. It has also been effectively examined how using varied PEG molecular weights can increase the bactericidal impact and it has been observed that PEG surface modification has enhanced metal nanoparticles antibacterial activity overall^97^. Furthermore, the longer PEG chains are preferable because more hydroxyl groups are formed on the surface of metal nanoparticles in longer-chain polymers, increasing their bactericidal activity. In addition, the structural characteristics of the higher-molecular-weight PEG, which has a greater affinity with actin proteins and can inhibit cellular processes in bacterial cells^98,99^. Additionally, higher molecular weight PEG may have greater antibacterial efficacy because of their high hydrophilic characteristics, which enable more water to be removed and inhibit microbial development because bacteria require a certain amount of water to grow optimally^100^. Hydroxyl groups of the PEG polymer chain could also weaken the extracellular polymeric substance—membrane attachment^101^ which would have a detrimental effect on bacterial growth. PEG's hydroxyl groups can also be used to form intricate nanostructured films with higher antibacterial activity than simple metal nanoparticles^102^.

According to our findings functionalizing the metal nanoparticles with PEG led to higher antibacterial activity. These functionalized nanoparticles could be utilized successfully in the future to coat food packaging, surgical instruments, and delicate devices due to their significant antibacterial activity. Growth curves were typically obtained by monitoring the optical density (OD), at the wavelength of 600 nm, a typical wavelength for cells. The density of bacterial isolates must be adjusted to an optimal density of 0.5 McFarland standards and the OD should serially be monitored hourly up to 12 h of incubation. Distinctive mechanisms that have been put forward in the literature are listed as follows: direct contact of ZnO nanomaterial with cell walls, resulting in destructing bacterial cell integrity^17,85,86^, liberation of antimicrobial ions mainly Zn^2+^ ions^72,103,104^, and ROS formation^105–108^. However, the toxicity mechanism varies in various media as the species of dissolved Zn may change according to the medium components besides the physicochemical properties of ZnO nanomaterials^72^.

As shown in Table 4, the antibacterial activity of the ZnO nanoparticles which showed the highest activity was compared with the antibacterial activity of ZnO reported in the literature. It is worth noting that the antibacterial activity reported here is greater in some studies and lesser in some other studies. The antibacterial activity is dependent not only on the type of nanomaterial but also on the size, shape and capping agent used during the synthesis. Further, antibacterial activity also depends on the experimental and environmental conditions.

**Table 4:** A comparison of the antibacterial activity of ZnO compared to the literature.

Nanomaterial	Concentration	Zone of inhibition		References
		*E.* *coli* (mm)	*Staphylococcus aureus* (mm)	
ZnO	10 mg/ml	Resistant	1.9	^109^
ZnO	10 mg/ml	19	29	^110^
ZnO	10 µg/ml	25	22	^111^
ZnO	N/A	4.96	–	^6^
ZnO	20 mg/ml	14.33	25.67	This study

### Antioxidant activity

The antioxidant activity of the nanocomposites synthesized was determined by DPPH (2,2-diphenyl-1-picrylhydrazyl) assay using 20 mg/ml, the lowest concentration used for the antibacterial study including for kill curves. In the presence of sunlight, all the nanocomposites generate electron hole pairs which lead to the generation of oxygen based free radicals where the electron density is transferred from the oxygen atom to the odd electron located at the N of the DPPH molecule resulting in the formation of the stable DPPH molecule converting the purple colour solution to yellow colour. The highest DPPH scavenging activity resulted in the presence of Cu:Zn 1:2 (51.13%) followed by ZnO (47.79%), Cu:Zn 1:1 (33.85%) and Cu:Zn 2:1 (32.06%). Free radical generation depends on the charge transfer mechanism resulting in the proper band alignment which also influenced the resulting results in the DPPH assay. The generation of radicals by the nanocomposites evident in this study further supports the existence of an antibacterial mechanism in which the radicals are involved in creating an inhibitory effect on bacteria.

Further, dye adsorption and degradation studies were also conducted. It was noted that no dye adsorption occurred for all the composites synthesized throughout the period tested. Moreover, the same dye samples were exposed to sunlight in the presence of the composites synthesized to investigate the photocatalytic activity and noticed that no dye degradation also occurred during the period in all samples though the generation of radicals was proven from the antioxidant study described above. The nanocomposites are *insitu* functionalized with PEG of which the steric hindrance is high. The surface of the nanocomposites is covered with PEG and hence bulky methylene blue molecules cannot reach the surface of the catalysts minimizing the adsorption. Though the radicals are generated, as the methylene blue molecules have not been adsorbed to the surface and are located away from the catalyst radicals do not reach the reactant molecules resulting in no dye degradation as observed.

The antioxidant activity resulted in this study was compared with the literature as given in Table 5. It is evident that though the studies given below haven’t used the same nanocomposite concentration, the antioxidant activities reported in this study for both the ZnO and CuO–ZnO are greater than the values reported.

**Table 5:** Comparison of the antioxidant activity.

Nanomaterial	Concentration	DPPH radical scavenging ability %	References
ZnO	12.5 mg/ml	22.66	^112^
	25 mg/ml	27.49	
	50 mg/ml	40.3	
CuO–ZnO	12.5 mg/ml	39.85	
	25 mg/ml	49.50	
	50 mg/ml	57.94	
Cu–ZnO	20 µg/ml	12.54	^113^
	100 µg/ml	59.45	
ZnO	20 mg/ml	47.79	This study
	40 mg/ml	54.11	
	60 mg/ml	55.07	
CuO–ZnO	20 mg/ml	51.13	
	40 mg/ml	40.52	
	60 mg/ml	74.49	

## Conclusion

PEG functionalized ZnO, Zn:Cu 2:1, Zn:Cu 1:1 and Zn:Cu 1:2 nanomaterials were fabricated by the co-precipitation method and used as antibacterial agents to inhibit the bacterial growth and/or kill the bacteria. The synthesized nanomaterial had inhibitory effects on Gram negative *Escherichia coli****,**** Pseudomonas aeruginosa*, *Klebsiella pneumoniae* and the Gram positive *Staphylococcus aureus* indicating their ability to penetrate both thin and thick peptidoglycan cell walls in agar well diffusion assay. Among the synthesized nanomaterials PEG-coated ZnO showed the highest antibacterial activity in inhibiting the growth of the above bacterial species, 14.33 ± 0.53, 15.17 ± 0.29, 12.00 ± 0.50 and 25.67 ± 0.58 mm, respectively with nanoparticle concentration of 20 mg/ml. The metal oxide nanocomposites were found to be more sensitive towards the Gram positive strain *Staphylococcus aureus.* Most Gram-positive bacteria have negatively charged teichoic acids (with substantial phosphate groups) that stretch from the cell wall to the surface which enhances the negative surface charge of their cell envelope allowing positively charged metal ions to bind more effectively. Additionally, the reactive oxygen species (ROS) produced in the presence of nanomaterials also can interfere with biological processes. All of the tested bacterial pathogen strains exhibited time-dependent rapid bactericidal action by nanomaterials, as shown by the time-kill synergy assay, which restricted the bacterial growth before they reached the stationary phase. The development and the growth of all the bacterial species were investigated in this study, restricted or slowed down by Cu:Zn 1:1 nanomaterial during the lag phase and log phase. Overall, the findings suggest that the synthesized nanomaterials are highly efficient in the lag and log phases against all the investigated bacterial pathogens. The antibacterial activity was further evaluated based on the MBC/MIC ratio and it was found that some of the synthesized nanomaterials possessed bacteriostatic effect while others possessed bactericidal effect on test organisms. The utilization of polyethylene glycol (PEG) as a coating agent, significantly increased the antibacterial activity of the fabricated antibacterial agents. The antioxidant activity of the synthesized nanomaterials varied as Cu:Zn 1:2 (51.13%) followed by ZnO (47.79%), Cu:Zn 1:1 (33.85%) and Cu:Zn 2:1 (32.06%). Overall, the fabricated biocompatible nanomaterials can be used as models to be used in biotechnological, pharmaceutical and food packaging applications owing to their high antibacterial activity, stability and durability. However, the main limitations before industrial implementation seem to reside in the need for carefully assessing any possible nano-toxicology effect of the fabricated nanomaterials, related to the exposure of eukaryotic cells and hence further research is required to assess the safety and the risk of these novel nanomaterials.

### Supplementary Information

Supplementary Figures.

## Data Availability

The datasets used and/or analyzed during the current study are available from the corresponding author on reasonable request.
