# Supplementary material for: In vitro influence of PEG functionalized ZnO–CuO nanocomposites on bacterial growth
Source: Sci Rep. 2024 Jan 14;14:1293. doi: 10.1038/s41598-024-52014-6 (PMC10788344; doi:10.1038/s41598-024-52014-6)
Supplement: Supplementary file 1 — Supplementary Figures. [file 41598_2024_52014_MOESM1_ESM.docx]

**In vitro influence of PEG functionalized ZnO-CuO nanocomposites on bacterial growth**

Madara Jayanetti^1^, Charitha Thambiliyagodage^1^, Heshan Liyanaarachchi^1^, Geethma Ekanayake^1^, Amavin Mendis^1^, Leshan Usgodaarachchi^1^

^1^Faculty of Humanities and Sciences, Sri Lanka Institute of Information technology, New Kandy Road, Malabe, Sri Lanka


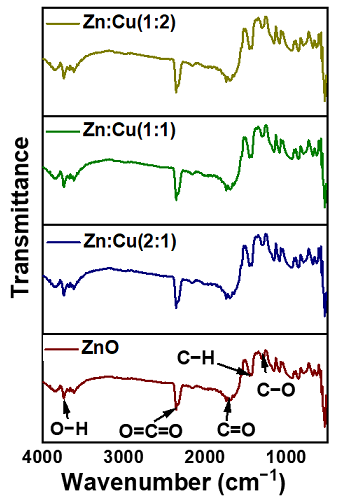


Figure 1. FT-IR spectra of the synthesized nanomaterials


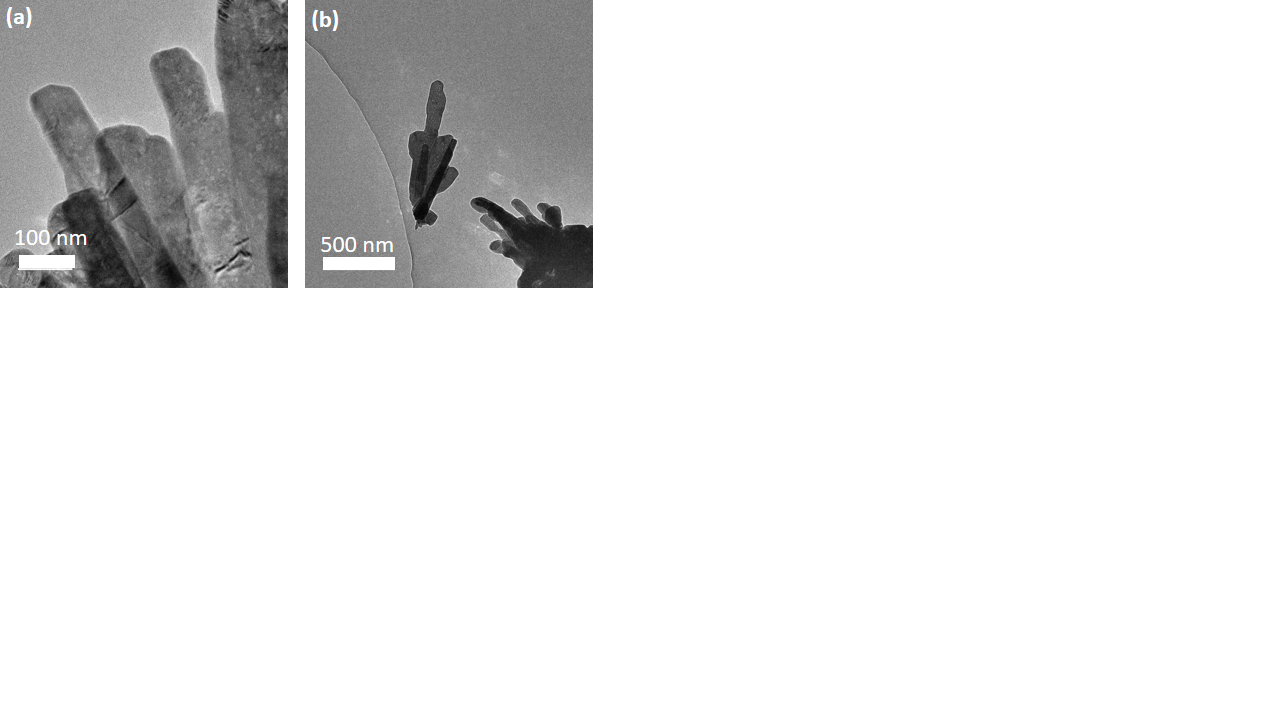


Figure 2. TEM images of (a) Zn:Cu (2:1) (b) Zn:Cu (1:1)
